# Large-Scale Determination of Sequence, Structure, and Function Relationships in Cytosolic Glutathione Transferases across the Biosphere

**DOI:** 10.1371/journal.pbio.1001843

**Published:** 2014-04-22

**Authors:** Susan T. Mashiyama, M. Merced Malabanan, Eyal Akiva, Rahul Bhosle, Megan C. Branch, Brandan Hillerich, Kevin Jagessar, Jungwook Kim, Yury Patskovsky, Ronald D. Seidel, Mark Stead, Rafael Toro, Matthew W. Vetting, Steven C. Almo, Richard N. Armstrong, Patricia C. Babbitt

**Affiliations:** 1 Department of Bioengineering and Therapeutic Sciences, University of California, San Francisco, San Francisco, California, United States of America; 2 Department of Biochemistry, Vanderbilt University School of Medicine, Nashville, Tennessee, United States of America; 3 Department of Biochemistry, Albert Einstein College of Medicine, Bronx, New York, United States of America; 4 Department of Biochemistry, University of Wisconsin, Madison, Wisconsin, United States of America; 5 Departments of Biochemistry and Chemistry, Vanderbilt University School of Medicine, Nashville, Tennessee, United States of America; 6 Department of Pharmaceutical Chemistry, University of California, San Francisco, San Francisco, California, United States of America; 7 California Institute for Quantitative Biosciences, University of California, San Francisco, San Francisco, California, United States of America; European Bioinformatics Institute, United Kingdom

## Abstract

The cytosolic glutathione transferase (cytGST) superfamily comprises more than 13,000 nonredundant sequences found throughout the biosphere. Their key roles in metabolism and defense against oxidative damage have led to thousands of studies over several decades. Despite this attention, little is known about the physiological reactions they catalyze and most of the substrates used to assay cytGSTs are synthetic compounds. A deeper understanding of relationships across the superfamily could provide new clues about their functions. To establish a foundation for expanded classification of cytGSTs, we generated similarity-based subgroupings for the entire superfamily. Using the resulting sequence similarity networks, we chose targets that broadly covered unknown functions and report here experimental results confirming GST-like activity for 82 of them, along with 37 new 3D structures determined for 27 targets. These new data, along with experimentally known GST reactions and structures reported in the literature, were painted onto the networks to generate a global view of their sequence-structure-function relationships. The results show how proteins of both known and unknown function relate to each other across the entire superfamily and reveal that the great majority of cytGSTs have not been experimentally characterized or annotated by canonical class. A mapping of taxonomic classes across the superfamily indicates that many taxa are represented in each subgroup and highlights challenges for classification of superfamily sequences into functionally relevant classes. Experimental determination of disulfide bond reductase activity in many diverse subgroups illustrate a theme common for many reaction types. Finally, sequence comparison between an enzyme that catalyzes a reductive dechlorination reaction relevant to bioremediation efforts with some of its closest homologs reveals differences among them likely to be associated with evolution of this unusual reaction. Interactive versions of the networks, associated with functional and other types of information, can be downloaded from the Structure-Function Linkage Database (SFLD; http://sfld.rbvi.ucsf.edu).

## Abstract 2

Global networks of the cytosolic glutathione S-transferases illuminate sequence-structure-function relationships across more than 13,000 members of this superfamily, including experimental confirmation of enzymatic activity for 82 members and new crystal structures for 27.

## Author Summary

Cytosolic glutathione transferases (cytGSTs) are a large and diverse superfamily of enzymes that have important roles in metabolism and defense against oxidative damage. They have been studied for several decades but because of the synthetic nature of the chemicals used to test these proteins to determine if they have cytGST activity, little is known about the physiological reactions and roles of cytGSTs. In this large, collaborative study, we constructed networks where more than 13,000 cytGST sequences were grouped by sequence similarity and then used these networks to prioritize new targets for experimental characterization in relatively unexplored regions of the superfamily. We report here experimental results confirming GST-like activity for 82 of them, along with 37 new three-dimensional molecular structures determined for 27 targets. These new data, along with experimental data previously reported in the literature, were painted onto the networks to generate a global view of their sequence-structure-function relationships. The results show how proteins of both known and unknown function relate to each other across the entire superfamily and illuminate the complex ways in which their variations in sequence and structure affect our ability to predict unknown functional properties.

## Introduction

The cytosolic glutathione transferases (cytGSTs), with several Enzyme Commission (E.C.) [1] numbers, principally 2.5.1.18, comprise one of four major groups of enzymes known to catalyze a general reaction involving nucleophilic attack by reduced glutathione (GSH) on compounds that contain an electrophilic carbon, nitrogen, oxygen, or sulfur atom (Reaction (1)) [2],[3].
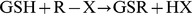
(1)Each group represents a different superfamily and/or structural fold; the cytGST superfamily described in this work is the largest of these four groups. The cytGSTs are soluble proteins that occur in a huge group of proteins that share overall structure similarity, the “thioredoxin-like fold” [4] that we hereafter refer to as the “thioredoxin fold.” The cytGST enzymes play many important roles in the cell, including metabolism of endogenous compounds, detoxification of xenobiotics, and defense against oxidative stress. Some of these proteins are of particular interest as targets of antiasthmatic and cancer drugs [2],[3],[5]. Especially in microorganisms, their functions are particularly diverse and largely unexplored. The other three groups of proteins that catalyze GST reactions include: (1) the mitochondrial GSTs (kappa class enzymes) [6],[7] that are members of a different and only distantly related superfamily of the thioredoxin fold; (2) the MAPEG (membrane-associated proteins in eicosanoid and glutathione metabolism) or “microsomal” GSTs [8],[9], and (3) members of the vicinal oxygen chelate (VOC) fold superfamily [10]–[12]. The latter two groups come from other, structurally distinct, fold classes.

The structures of the cytGST superfamily are defined by a minimum requirement for two domains, typically ordered as a conserved N-terminal domain of the thioredoxin fold that includes the glutathione binding site, and a C-terminal domain that is of a different fold and that is more diverse across the members of the superfamily as it provides the binding site for the many different substrates that these enzymes can turn over. Although both domains are required for activity and are fused in a single polypeptide, the fold-centric SCOP [13] and CATH [14] classifications, and the Pfam [15] resource classify these as separate N- and C-terminal domains. Large-scale structural comparison shows that the cytGSTs are divergent from other superfamilies of the thioredoxin fold with the N-terminal domain easily distinguished from those other superfamilies [16]. Divergent sequences homologous to the cytGSTs whose functions differ from those of the cytGST enzymes have been identified [17] and will likely continue to be discovered. Some of these “outlier” proteins, for example, the chloride intracellular channel proteins (CLICs) [18], are included in our dataset (see Methods for details on construction of the dataset) but their functions are not a focus of this analysis and so are not discussed in this paper.

The many studies describing the sequences, structures, and functions of the cytGST superfamily proteins have led to their classification into a number of distinct classes, largely reflecting sequence similarity and, for more closely related sub-classes, knowledge about substrate similarities or other functional features [5],[19],[20]. Historically, cytGSTs were grouped into classes designated by Greek letters (e.g., “Alpha,” “Mu,” “Pi,” “Sigma,” etc.), with new classes added as they were discovered. Each class was originally defined by the first researchers to study its members, then refined over several decades as new cytGSTs were characterized. Broader studies of subsets of cytGST classes have been published [20]–[22] and provide in-depth discussion about many issues, including findings of structural similarity among members and how to standardize nomenclature. However, no systematic rationale for an overall division of cytGST sequences, whether of known or unknown function, is available for classification of the entire superfamily. There has also never before been an attempt to correlate reaction types with sequence- and structure-similarity relationships over the entire superfamily.

The UniProtKB/Swiss-Prot resource (hereinafter called “Swiss-Prot”) [23] has compiled much of the available classification information for the experimentally characterized cytGSTs, along with new sequences their curators have assigned to these classes on the basis of sequence similarity. We term this compilation “canonical” classes to distinguish them from the new subgroups of sequences proposed in this work. Because it provides a high quality representation of available knowledge about cytGST functions that can be accessed on a large-scale using automated methods, the Swiss-Prot classification was used as the main source of the canonical classification data used in this paper. Two additional classes are not yet included in Swiss-Prot; the Nu [24] and Xi [25],[26] classes have been recently defined in the literature and are also included as canonical classes in this study.

Despite the attention that cytGSTs have received, classification of their reactions and biological roles has remained challenging for many reasons. First, the further addition of new classes and of new proteins to existing classes has typically been done in an ad hoc manner as each new divergent group was discovered. This has resulted in the definition of classes of varying granularity where better studied groups have tended to have more numerous and smaller classes defined, complicating our understanding of their similarity relationships. Second, large numbers of cytGST-like sequences of unknown function have now been identified that do not belong to any of the canonical classes. Only recently have relationships been determined among some of these groups on a larger scale [20],[27]. Third, even for experimentally characterized cytGSTs, classification is still complicated because of their ability to catalyze a wide range of chemical reactions and their tendency to show substrate specificities that broadly overlap even across quite different classes. Moreover, few of the natural substrates of GSTs are known, so that the substrates used to define or discriminate cytGST-catalyzed reactions are often synthetic compounds. A primary example is 1-chloro-2,4-dinitrobenzene (CDNB), a substrate that can be turned over by many different GSTs. With the advent of new technology along with enormously increased information about these proteins in public databases, a more systematic analysis of sequence and structure relationships is timely.

To begin to address these challenges and aid in functional inference for the majority of proteins of unknown function that now populate the superfamily, we performed all-by-all similarity comparisons among approximately 13,000 nonredundant cytGST-like sequences. The results can be viewed and explored in the form of graphical network models called protein similarity networks [28]. In these networks, sequences or structures are grouped by similarity to summarize their relationships on a large scale. The subgroup partitions that result can then be mapped with functional assignments and many other types of biological information to identify functional trends from the context of those sequence and structural relationships.

In the networks shown in this paper, nodes (circles, other shapes) represent sequences and/or structures, and edges (lines between nodes) represent sequence or structure similarities with scores better than a chosen statistical significance threshold. Node properties (size, color, etc.) are then used to visualize functional or other information mapped to the networks. Descriptions of the creation and validation of thresholded protein similarity networks, along with some of their uses and limitations, have been reported elsewhere [28],[29]. The networks can be visualized and interactively explored using commonly available software such as Cytoscape [30]. Because very large networks (more than a few thousand nodes and their associated edges) cannot be visualized on commonly used lab computers, most of the networks provided in this paper are “representative” networks [29] in which each node represents one or more member sequences that all share a user-defined percent identity (at least 50% identity in this work). For these compacted networks, edges between any two representative nodes are drawn if the least significant pairwise similarity score between the representative sequences is better than the statistical significance cutoff used to visualize that network. Full networks, in which each node represents a single sequence, provide a more detailed view of specific subgroups.

We used the networks to guide our choice of sequence targets to sample broadly across the superfamily putative cytGSTs of unknown function. The targets were then processed using a high-throughput pipeline to express and purify them, and, as possible, determine crystal structures of the purified proteins recovered from the pipeline. We report here new experimental results confirming cytGST-like activity for 82 enzymes that had not previously been characterized, along with the determination of 37 new structures. These results, along with experimentally known GST reactions and structures reported in the literature, were mapped to the networks to generate a global view of their sequence-structure-function relationships across the superfamily. (More detailed reports of these experimental results than can be addressed here will be presented in related papers.)

The results show that the great majority of sequences assigned to the cytGST superfamily have not been experimentally characterized or even assigned to one of the canonical classes. Moreover, this global view shows for the first time how proteins of both known and unknown function relate to each other across the entire superfamily, providing a foundation for an extended classification system that includes the many unknowns revealed by this study and new clues for their functional inference.

The remaining sections highlight other broad themes about sequence-structure-function relationships in the cytGSTs, many of which had been observed only for individual or small groups of superfamily members. The first describes a mapping of taxonomic representation across the superfamily, revealing its complex nature. Next, we discuss a hallmark characteristic of the cytGSTs, occurrence of the same reaction type in many different subgroups. Finally, we provide a closer look at sequence-structure-function relationships in a subgroup that includes an unusual cytGST reaction, illustrating nature's use of GST chemistry to evolve specialized reactions quite different from those associated with their roles in main metabolism.

## Methods

### Construction of Sequence Similarity Networks and Designation of Subgroups

All networks were visualized using Cytoscape (v2.8.3) [30], where each node represents a protein or group of proteins and an edge or line between the nodes denotes a similarity relationship between the proteins. The “organic” layout was used whereby nodes are clustered more tightly if they are more highly interconnected. Although edge lengths in this layout are not explicitly correlated in the organic layout with similarity distance, previous work has shown that the relative distances calculated by this layout are close to the mathematically ideal BLAST *E*-value distances (see supplementary material in Atkinson et al. [28]).

To construct sequence similarity networks, 13,435 full-length sequences that were at least 100 residues in length were taken from Pfam (v26.0) [15] that had scores above the Pfam gathering threshold for at least one hidden Markov model (HMM) for the conserved thioredoxin fold domain, i.e., the cytGST N-terminal domain (collectively referred to here as “GST_N*”). Fifty-eight additional proteins identified by sequence similarity to cytGSTs were also added. The entire set of 13,493 non-redundant proteins is referred to here as “cytGSTdb”; these sequences were deposited in the Structure-Function Linkage Database (SFLD).

Two types of protein similarity networks were constructed: full networks and representative networks. First, all-by-all BLAST comparisons of all of the sequences in cytGSTdb were performed using blastp (v2.2.24; default settings except with seg filtering turned off) [31]. For full networks, the *E*-value for the highest BLAST score between each protein pair was used in generating network edges between nodes; each node depicts a single sequence. To reduce noise and allow visualization of subgroupings depicting our results in the networks, they are thresholded using an *E*-value cutoff so that nodes are connected only if the *E*-value of the relevant BLAST score is better than the cutoff threshold (see Atkinson et al. [28] for a discussion of thresholded networks). Because visualization of protein similarity networks using Cytoscape is limited by the number of edges in the network, full networks cannot be opened and explored interactively for most of the level 1 and many level 2 sequence similarity networks provided. Instead, we used representative sequence similarity networks, as described in the main text and relevant supporting information.

Representative networks are composed of abstracted nodes and edges, with each node representing one to many sequences binned with a percent identity filter. To generate representative networks for this study, CD-HIT [32] was used to filter the sequences to 50% sequence identity (ID50), with one sequence chosen as the representative for each ID50 node. To achieve our goal of capturing the greatest possible detail of the sequence space of the cytGSTs, we used the least stringent percent identity cutoff that would both include the greatest number of sequence representatives and maintain the ability to view and manipulate the networks in a reasonable amount of time on our computers. This resulted in 2,190 ID50 nodes representing the 13,493 member sequences in cytGSTdb. The similarity score between each pair of representative nodes corresponds to the least significant BLAST *E*-value between the representative sequences. Representative nodes were annotated to a cytGST class or taxonomic category if >50% of its annotated members had that annotation. Taxonomy classification was assigned using NCBI taxonomy. Reaction types were assigned to representative nodes if any member sequence in that node had experimental evidence for that reaction type.

Class annotations were from Swiss-Prot [23],[33]; some additional annotations were obtained from literature references for the recently described Nu [24] and Xi [25],[26] classes. Swiss-Prot is a manually curated database of protein sequences shown to have among the most reliable annotations that can be obtained from large online databases [34]. Reaction types were assigned according to experimental evidence from this work and the literature.

The *E*-value thresholds chosen for visualization of the representative sequence similarity networks were determined by stepping through *E*-value thresholds until the first major separation of clusters (groups of interconnected nodes separated from other interconnected nodes) appeared and persisted through several more thresholds. Clusters at a threshold of 1×10^−13^ were designated as level 1 subgroups (e.g., “Main,” “R1”).

To choose the level 2 threshold, further thresholds were stepped through and visually examined. It was observed that at an *E*-value cutoff of 1×10^−25^, good separation of canonical classes occurred. At more stringent thresholds, there was even better canonical class separation, but it was also seen that annotated nodes began to separate from others of the same class. To avoid losing information in the network views depicting how nodes of the same class relate to each other across the sequence space, the less stringent level 2 threshold was chosen as a compromise. This allowed good separation by canonical class while keeping together most nodes annotated to the same canonical class; i.e., grouping nodes together with others of their historically assigned class was favored over separation. At this threshold (1×10^−25^), clusters were designated as level 2 subgroups under their level 1 subgroup parent (e.g., “Main.1,” “R1.1”). For both level 1 and 2, clusters with at least 50 member sequences were designated as subgroups. For creation of full networks (in which each node represents a single sequence), all of the member sequences associated with each representative node were assigned to the same subgroup as their representative node (and are also designated this way in the SFLD).

To view the details of each level 2 subgroup, a full network was generated for each. Here, where each node represents a single sequence, a more stringent threshold was used to create and view clusters that are better separated according to canonical class assignments. To help guide the choice of threshold for these full networks, we applied the Markov Cluster Algorithm (MCL) [35], an objective method that has been used to detect protein families [36], to the representative network data. We then used our own metric for calculating the *E*-value threshold (1×10^−31^) at which network clusters best correlated with both canonical class assignments and MCL clusters (see Text S1 for details). A full network for each level 2 subgroup was constructed using all member sequences in the previously defined subgroups and visualized at the 1×10^−31^ cutoff. Visual review of these groupings confirmed that the groupings at this threshold looked reasonable with regard to annotated classes. Two of these full networks are depicted in this paper and all of the full networks generated in this manner for every level 2 subgroup can be downloaded from the SFLD.

### Structure Similarity Networks

To generate structure similarity networks, we used the SFLD to identify 392 crystal structures for cytGSTs in the Protein Data Bank (PDB) [37] with ≥90% sequence identity to one or more of the 13,435 cytGST sequences in our dataset. Six additional structures were obtained from the Structure Core of the Enzyme Function Initiative (EFI) [38] prior to completion of their deposition in the PDB. Of these 398 structures, 37 were solved by the EFI and a pilot study for the EFI within the New York SGX Research Center for Structural Genomics (NYSGXRC, PSI-II) [39]. The EFI has a Protein Core Facility that produces protein for each target. These proteins were shipped to the Structure Core Facility for structure determination and to the Armstrong laboratory for activity assays. Because structures may have missing domains, sequences for the 398 structures were extracted from the PDB ATOM lines and searched with the GST_N* models using HMMER (v2.3.2, gathering threshold) [40]. For the 379 structures with an HMM match, pdbaa chain sequences were filtered to 95% sequence identity (ID95) with CD-HIT, and representative structures for each ID95 group (131 total) were chosen, taking into consideration qualities such as resolution. After trimming extraneous domains, 3D structure similarity was calculated from all-by-all pairwise comparisons using FAST [41], and the representative networks were visualized with Cytoscape. Class and reaction type annotations were assigned according to the annotations for the sequences associated with each PDB structure.

### Experimental Assays and Methods

Guided by sequence similarity networks, targets for experimental characterization were chosen to cover sequence space broadly, with proteins for which little was known about their structures and functions and for which DNA was readily available prioritized for characterization. Sequences from prokaryotes were preferred because of ease of protein production and to allow microbiology studies. Purified proteins were obtained from SGX Pharmaceuticals (SGX), which supplied protein for NYSGXRC [39] or the Protein Core of the EFI. Proteins were expressed and purified as previously published [39],[42]. Activity for the targets for which purified protein was available was determined in a high-throughput assay scheme using known cytGST substrates. This scheme utilizes a 96-well plate reader formatted either for single time point or continuous assays. The conditions for each assay were individually optimized. The substrates used and relevant references to the primary literature are provided in Table S1. More detailed assay results including kinetic information will be described in related papers in preparation. Data on EFI protein targets and their progress regarding stage of expression, purification, and crystallization may be found at EFI-DB, the EFI public database of experimental data (http://kiemlicz.med.virginia.edu/efi/).

### Structures

The details of the crystallization and structure determination will be discussed in future publications. Briefly, crystals were obtained by the sitting-drop vapor diffusion method. X-ray diffraction data were collected at 100°K on either beamline X29A (National Synchrotron Light Source, Brookhaven National Laboratory, Upton, New York) or at the beamline 31-ID (wavelength of 0.979 Å, Advanced Photon Source, Chicago, Illinois). Structures were determined by Se-MET SAD (typically those crystals originating from SGX sourced protein) or by molecular replacement (those crystals originating from EFI sourced protein). All solved structures were deposited in the PDB.

### Compilation of Experimental Evidence for GST Activity for Proteins Previously Annotated to Have GST Activity

The gene_association.goa_uniprot file from GOA [43] (a database of evidence-based associations between Gene Ontology [GO] [44] terms and UniProt proteins) was parsed to identify proteins experimentally assayed for GST-like activity. Because substrates are not included in the GOA information, that information was obtained manually from literature referenced by GOA. Also, the manually curated databases of biochemical reactions BRENDA [45] and SABIO-RK [46] were mined for data corresponding to the following GST-relevant E.C. numbers: 2.5.1.18, 4.5.1.3, 5.3.99.2, 1.8.5.1, 1.11.1.9, 5.2.1.2, 5.2.1.4, 1.20.4.2, and 1.5.4.1. In addition, a manual search of the literature was performed. Experimental evidence for cytGST-like function was collected for 95 proteins from BRENDA, 58 from GOA, 35 from the manual literature search, and 17 from SABIO-RK for a total of 176 non-redundant proteins.

### Construction of Multiple Sequence Alignments

Multiple sequence alignments (MSAs) were constructed using ProbCons [47] or PROMALS3D [48],[49], a program that incorporates 3D structure into the calculations to help guide the alignment. Sequences were used if there was experimental evidence for disulfide bond reductase (DSBR) activity; their available cognate structures were used if they did not have engineered mutations. In order to help guide the alignment, two structures without DSBR evidence were included from subgroups lacking 3D structures with DSBR evidence. Alignments were visualized with Jalview [50].

## Results and Discussion

**Figure 1:**
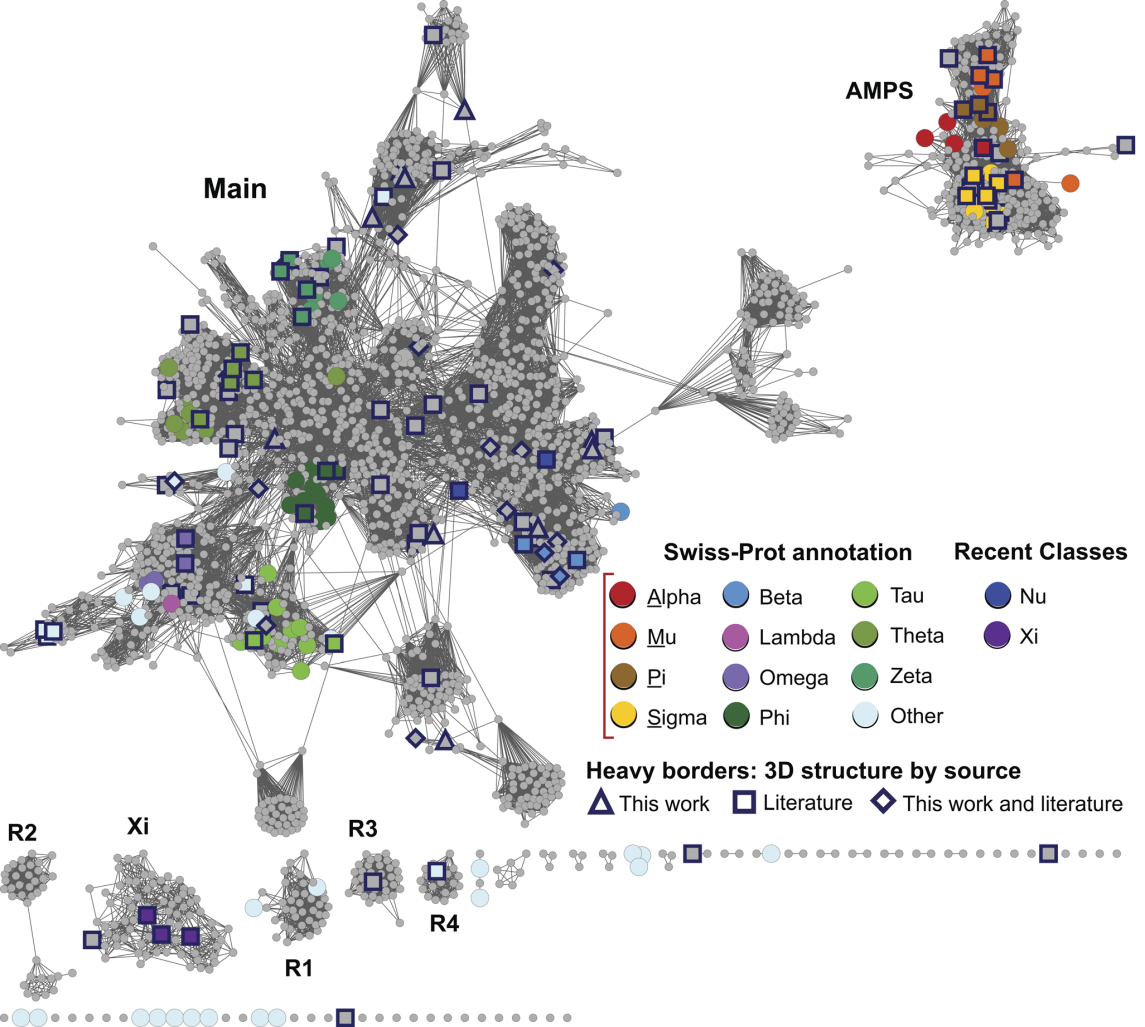
Global view of sequence relationships in the cytGST superfamily. This level 1 representative network shows 2,190 nodes representing 13,493 proteins filtered at 50% sequence identity. A cluster (a group of interconnected nodes separated from other groups of interconnected nodes) is labeled if there are at least 50 member sequences in that cluster. These include the large and diverse Main subgroup, the AMPS subgroup containing the Swiss-Prot classes Alpha, Mu, Pi, and Sigma, the recently described Xi subgroup, and several smaller but distinct clusters labeled R1–R4. Colors assigned correspond to Swiss-Prot annotations for canonical cytGST classes or to annotations from the literature for the newer classes Nu and Xi. These representative nodes are colored only if at least 50% of Swiss-Prot annotated sequences in that node have been assigned to that class. Grey nodes denote representative nodes for which no corresponding Swiss-Prot annotation is available for a class for greater than 50% of the annotated sequences in that node. Heavy borders indicate that a 3D crystal structure is associated with at least one member sequence of a representative node and shape indicates the source of the structure data: triangle, structures that were solved for this work; square, from the literature; diamond, structure evidence both from this work and the literature. Edges or lines between nodes are shown if the least significant pairwise sequence similarity score between the representative sequences of two nodes is better than the threshold (BLAST *E*-value≤1×10^−13^). The 32,716 edges depicted have a median percent sequence identity of 33% over 208 residues.

**Figure 2:**
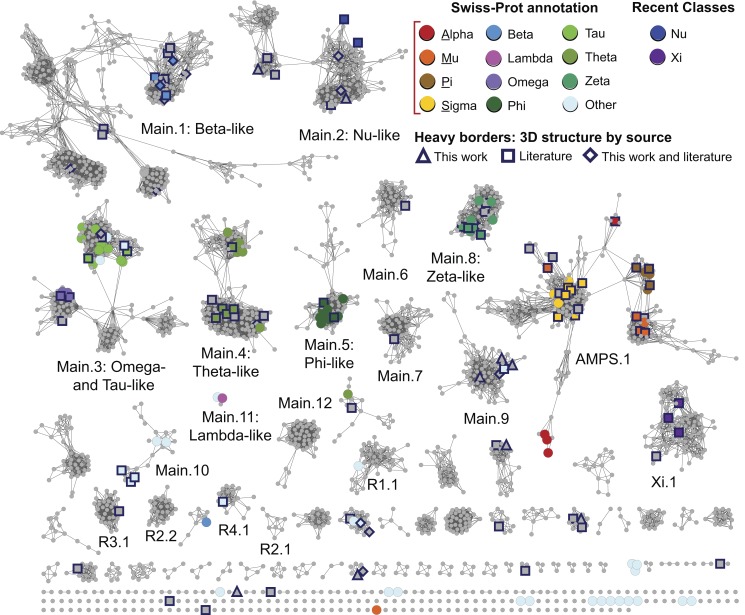
The level 2 representative network shows more detailed subgroupings. The same network as in Figure 1 except that it is visualized at a higher stringency threshold, i.e., *E*-value≤1×10^−25^. Coloring is the same as in Figure 1. The 15,070 edges depicted in the figure have a median percent sequence identity of 38% over 212 residues. As with level 1 subgroups, clusters are designated as level 2 subgroups if there are at least 50 member sequences in that cluster.

**Figure 3:**
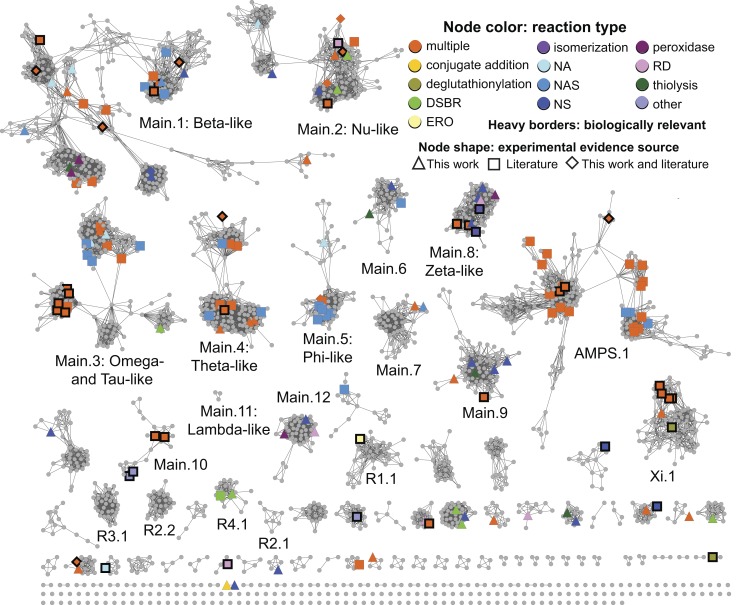
The level 2 representative network painted with known reaction type. Nodes are colored if at least one member in a representative node has experimental evidence for that reaction type. Colors denote reaction types as given in Figure 4, with the additional category of multiple reaction types (“multiple”), where orange indicates more than one reaction type occurring in a node. Some reaction types in Figure 4 are not represented by a separate color because they are subsumed by the “multiple” category. Single reaction type abbreviations: DSBR, disulfide bond reductase; ERO, epoxide ring opening; NA, nucleophilic addition; NAS, nucleophilic aromatic substitution; NS, nucleophilic substitution; RD, reductive dehalogenase. Node shapes indicate the source of experimental evidence for each reaction type: triangle, this work; square, literature; diamond, from this work and the literature. Nodes with member sequences that have evidence for biologically relevant reactions/functions are marked with thick black borders.

In the sections below, we describe a large-scale analysis of sequence, structure, and functional relationships among cytGSTs, using similarity networks to provide a global context for interpreting them. A level 1 network (Figure 1), which maps literature-defined classes (canonical classes) to the sequences that have been previously assigned to them, shows that the majority of cytGSTs are unknowns. Division of the global level 1 network into 35 subgroups allows more detailed views (level 2 networks) (Figure 2) of these relationships and provides a foundation for assignment of unknowns either to extant classes or to new classes suggested by these subgroupings. (See Table S2 for counts of representative nodes and member sequences in these subgroups.) Since the canonical classes describe only general functional or other properties resulting from previous experimental studies (or inferred by homology to founder members of a class), we also provide a version of the level 2 network to which experimentally determined cytGST reaction types have been mapped (Figure 3). This network view includes a mapping of new experimental results obtained in this study that confirms cytGST-like function for 82 unknowns across the superfamily sequence space and indicates on the networks where the 37 new structures for 27 protein targets occur. (See Table S3 for experimentally determined cytGST reaction types in representative nodes, Table S4 for the substrates of experimentally determined cytGSTs, and Table S5 for a full list of cytGST structures mapped onto the networks described in this study.)

The full diversity of a set of sequences or structures in a superfamily or other large group of proteins is often termed “sequence space” or “structure space.” These new results expand the coverage of our experimental knowledge of cytGST reaction types to many unexplored regions of their sequence and structure space, especially those for which no functional information has previously been available. The final sections highlight other characteristics of cytGSTs leading to new insights regarding how the cytGST fold has diverged to produce new functions.

Interactive versions of sequence similarity networks described in this work, associated with functional and other types of biological information, are freely available and can be downloaded from the SFLD [51],[52].

### A Global View of Sequence Relationships Shows the Majority of cytGSTs Are Unknowns

To summarize the coverage of the cytGST superfamily by the canonical classes and determine the distribution of unknowns, we compared more than 13,000 nonredundant sequences of the superfamily in an all-by-all manner. Figure 1 provides a global view of these relationships. Painting these networks by known assignments to canonical classes shows that the majority of the sequences are unknowns that have not previously been characterized or even assigned to canonical classes. The sequence-similarity based separation of these sequences using an *E*-value threshold of 1×10^−13^ (as described in Methods) into the labeled subgroups is shown in Figure 1. These are designated here and in the SFLD as “level 1 subgroups.”

The two largest subgroups in Figure 1 reflect a natural separation among cytGSTs based on sequence similarity. Some major differences among proteins in these two subgroups have been previously observed by many workers studying individual or groups of these enzymes. One of these subgroups constitutes proteins from the relatively well-studied Alpha, Mu, Pi, and Sigma classes (AMPS). The largest subgroup in the superfamily is designated as “Main” in this work and in the SFLD. The former includes mostly eukaryotic cytGSTs, including some heavily studied human enzymes, while the Main subgroup includes most of the other defined classes. Differences in these two large subgroups have been associated with differences in the position of the sulfur atom involved in GSH-assisted catalysis and with the amino acid residues in these enzymes that typically interact with it [27]. A third subgroup labeled in Figure 1 designates proteins of the recently described Xi class [25]. On the basis of the work reported here, four other subgroups, R1, R2, R3, and R4 (for “Remainder”) are also designated. In the SFLD, sequences not assigned to any of the labeled subgroups are assigned only to the superfamily but not to any subgroup within it.

#### Global analysis of the cytGST superfamily suggests few members are combined with additional domains

Analysis of the thousands of sequences in the cytGSTs provides an opportunity to estimate the proportion of member sequences likely to occur in combination with other domains. The cytGSTs investigated in this study range from 100 to 4,512 residues in length. Using length as a proxy to provide a rough estimation of the domain organization of the sequences in this superfamily and the proportion likely to contain additional domains not required for GST-like activity, we generated a histogram (Figure S1) showing the lengths of cytGSTs in our dataset. The histogram shows that the majority (71%) of these sequences fall into a length range of ∼190–275 residues, consistent with the size of biologically active cytGSTs. This conclusion is broadly consistent with Pfam predictions for the architectures of proteins in the cytGST superfamily. Only two other much smaller peaks occur in the histogram. In the first peak, comprising sequences of ∼300–350 residues in length, most were found to have two instances of the GST thioredoxin fold domain. Most of the sequences in the other peak, comprising sequences of ∼400–430 residues in length, have an instance of a translation elongation factor EF1B domain. For those cytGST proteins that do exist in combination with other domains, those additional domains, to the degree that they are similar to each other, could contribute to the BLAST similarity scores linking those proteins. However, similarity among the highly conserved and essential cytGST N-terminal (thioredoxin fold) domains present in all the sequences in the dataset dominates the relationships illustrated in the networks.

### A More Detailed Subgrouping Suggests Additions to the Canonical Classification System to Enable Classification of Most cytGST-Like Unknowns

To provide a more detailed view of sequence similarity relationships across the superfamily, we further subdivided the level 1 subgroups by requiring a greater degree of sequence similarity for drawing edges between nodes. These are designated as “level 2 subgroups” and are shown in Figure 2. Like the organization of the canonical classes, these level 2 subgroups reflect groupings based on sequence similarity, but unlike historical classifications, the sequence similarity grouping is based on a threshold that is applied uniformly and globally across the entire superfamily. This establishes a consistent framework for defining new classes that could be used to extend the current classification system to include the majority of unknowns that now populate the superfamily.

We attempted to choose a level 2 threshold that would distinguish similarity groupings at approximately the same granularity as the existing Swiss-Prot classes. The *E*-value threshold choice of 1×10^−25^ was a compromise for defining groupings that reflect good separation by canonical class, while also keeping together most annotated class nodes of the same class; grouping nodes together with others of their historically assigned class was favored over separation. (See Methods and Text S1 for more detail regarding how threshold choices were made.) These level 2 subgroups are named to reflect their level 1 parents. “Main.1” through “Main.12” are labeled in Figure 2; these represent the largest subgroups from the level 1 “Main” parent subgroup, with the exception of Main.11. Even though it designates a small set of only three representative nodes totaling 59 member sequences, the Main.11 subgroup is included in the top 12 “Main” list because it has a specific Swiss-Prot class annotation (Lambda).

There are 28 level 2 subgroups that arise from level 1 Main. In contrast, as can be seen in Figure 2, the membership of AMPS.1, Xi.1 (and some of the remainder subgroups designated in Figure 2 with an “R” label to distinguish them from the “Main,” “AMPS,” and “Xi” subgroup sets) are mostly unchanged from their parent level 1 subgroups. Over the entire superfamily, we defined 35 level 2 subgroups and 128 singleton representative nodes that are sufficiently diverse to be unconnected to any other nodes at this *E*-value threshold. Table 1 provides summary information about the representative and member sequences in the larger subgroups; full counts are provided in Table S2.

**Table 1:** Summary counts of representative nodes and member sequences in level 1 and level 2 subgroups.

Level 1 Subgroup	Level 2 Subgroup	Number of Representative Nodes	Total Number of Sequences
**AMPS**		262	1,033
	**AMPS.1**	205	921
**Main**		1,630	10,884
	**Main.1: Beta-like**	320	1,965
	**Main.2: Nu-like**	139	1,642
	**Main.3: Omega- and Tau-like**	154	1,252
	**Main.4: Theta-like**	149	872
	**Main.5: Phi-like**	58	422
	**Main.6**	38	191
	**Main.7**	51	338
	**Main.8: Zeta-like**	62	666
	**Main.9**	68	412
	**Main.10**	22	281
	**Main.11: Lambda-like**	3	59
	**Main.12**	48	417
**R1**		40	87
	**R1.1**	36	81
**R2**		37	193
	**R2.1**	8	97
	**R2.2**	25	92
**Xi-like**		80	920
	**Xi.1**	79	919

The counts for only the largest level 1 and level 2 subgroups are shown except for level 2 subgroup Main.11, as described in the text.

AMPS, Alpha-, Mu-, Pi-, and Sigma-like.

For each level 2 subgroup, we additionally constructed a full network, where, in contrast to representative networks, each node corresponds to a single sequence and all member sequences of a subgroup are used. Examples of full networks are provided in subsequent sections of this paper and full networks for all subgroups are available from the SFLD and are provided at this cutoff. These networks may also be easily viewed with different user-defined thresholds.

We do not suggest that the level 2 subgroups or the subclusterings visualized in the full networks be used to replace canonical classes or to rigidly define new classes. Instead, we propose that both the representative networks and the full networks provide useful guides for addressing inconsistencies from the ad hoc assignment of sequences to named classes. Further, they may serve as a starting point for the addition of new classes to the canonical classification system.

Only a minority of sequences (280 of the total 13,493 nonredundant sequences in the superfamily) have canonical class annotations either from Swiss-Prot or from the literature for the more recently described Nu [24] and Xi [25],[26] classes. Only 176 of the 13,493 sequences represented by the 2,190 representative nodes shown in Figure 2 had been experimentally confirmed to catalyze a GST-like reaction prior to this study, as far as can be inferred from online databases or our manual survey of the literature (see Methods).

### New Experimental Evidence Increases Confirmation of GST-Like Activity and Structural Coverage over the Breadth of Sequence Space in the cytGST Superfamily

**Figure 4:**
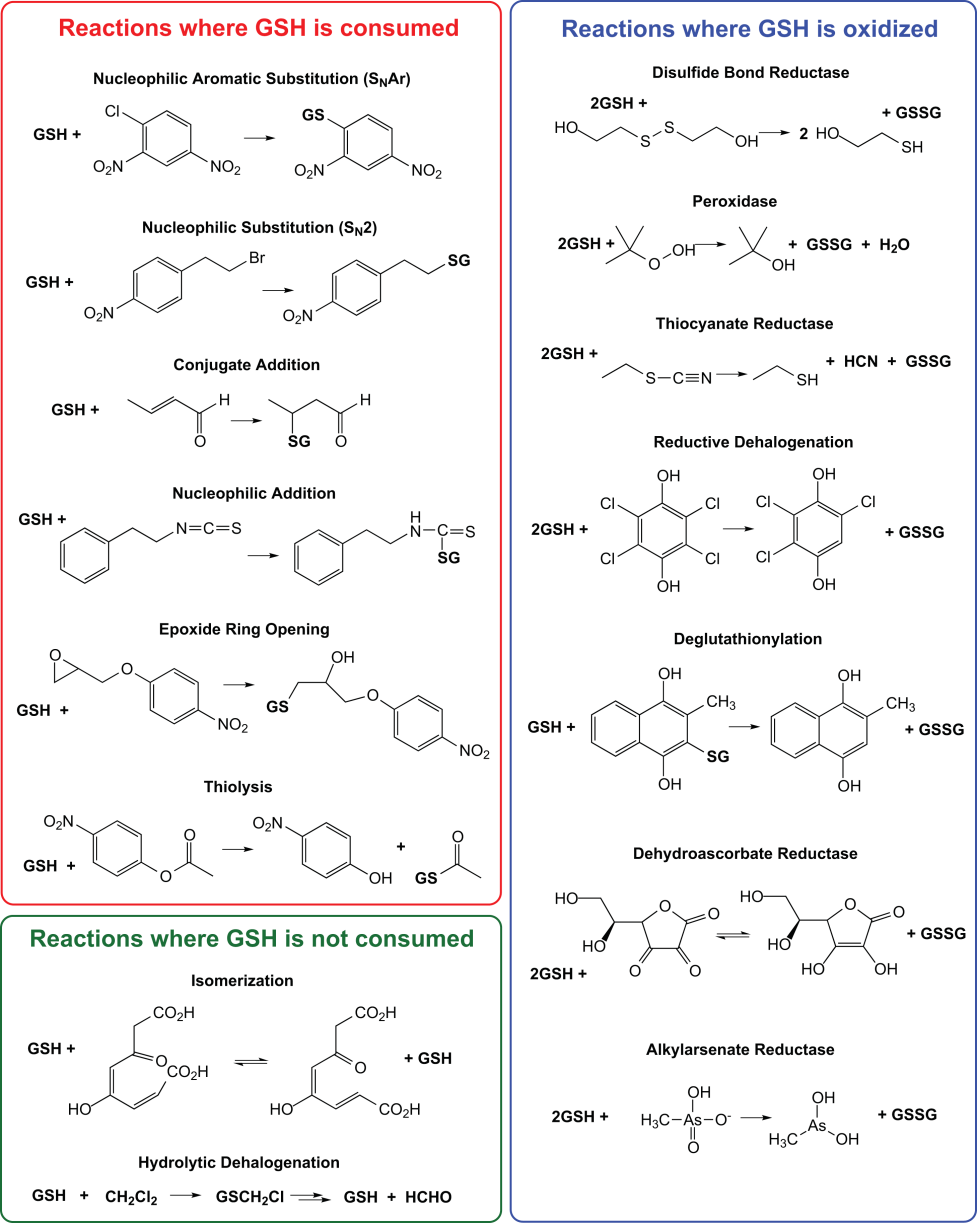
Major reaction types of the cytGST superfamily. Reactions are grouped by chemistry with a sample reaction shown for each reaction type.

Figure 3 shows the same network as in Figure 2, here mapped with experimental evidence for GST-like reaction types obtained both from assay results reported in this study and those we found from previous reports. (We cannot be sure that we found all of the annotations that have been published.) Figure 4 provides a listing of major GST reaction types where we have grouped reactions by similarity of chemistry. These 15 reaction types describe major known classes of GST-like reactions plus a category “other.” The “other” category represents some biologically relevant reactions/functions such as hematin binding [53] and pyrimidodiazepine synthase activity [54] (a key step in eye pigment formation in *Drosophila*) that differ from the general reaction types listed in Figure 4.

For experimental characterization, there were a total of 857 targets chosen from 31 level 2 subgroups and 64 smaller clusters/singletons not assigned level 2 subgroup names (see Methods). Proteins for approximately 230 targets were successfully purified for this work and assayed for GST-like activity. The new experimental evidence reported here confirms GST-like activity in 82 of these proteins that had not previously been assayed for this activity. These proteins occur in 20 level 2 subgroups and also in five smaller clusters (Figure 3); these new data account for an increase in the total number of clusters with experimental evidence for GST-like reactions from 23 to 35; seven level 2 subgroups lacked such experimental data before this study. Although these new results increase by about 50% the number of annotated proteins for which experimental evidence of cytGST-like activity is now available, the proportion of superfamily members that have been experimentally characterized with respect to reaction specificity remains small. Table S3 provides a summary of reaction types that occur in representative nodes in Figure 3. A full listing of cytGSTs that have experimental evidence for cytGST-like reactions, including UniProt accession numbers, available substrate information, and literature references, is in Table S4.

Figure 2 shows the coverage on the level 2 subgroups of the 37 new structures (for 27 new protein targets) determined by this work, along with previously determined structures. These new structures reported here expand the structural information available for cytGSTs substantially, especially for the Main.9 subgroup and several of the smaller level 2 subgroups. (Table S5 lists structures incorporated into this and other network views.)

The new structures solved in this work occur in 14 level 2 subgroups and in a representative cluster that is a singleton with only one member node (UniProt A5E0V2, PDB code 4EXJ). This sequence is less than 25% identical to any other sequence in the Main level subgroup in the SFLD as well as any other sequence in Genbank, making it especially divergent relative to the rest of the superfamily. Although none of the level 2 subgroups lacked structures prior to this work, the new structures reported here add significant data to the unexplored sequence space. Their contribution may be somewhat difficult to appreciate from the representative network shown in Figure 2 because of the compression of the information required to show relationships across the more than 13,000 sequences of the superfamily. This contribution is more readily apparent when viewing the full networks. Pre-existing structures populated 22 level 2 subgroups as well as some smaller clusters.

### Structural Comparisons Reveal Relative Connectivity among Subgroups and Support Relationships Inferred from Sequence Similarity Networks

While the level 2 networks (Figures 2 and 3) distinguish subgroupings useful for extending classification of cytGSTs, these assignments reveal little about which subgroups are most related to each other. As structural comparisons can capture more diverse relationships than can sequence comparisons [55], we generated structure similarity networks to complement the networks generated from sequence similarity and reveal relationships between subgroups in more detail.

**Figure 5:**
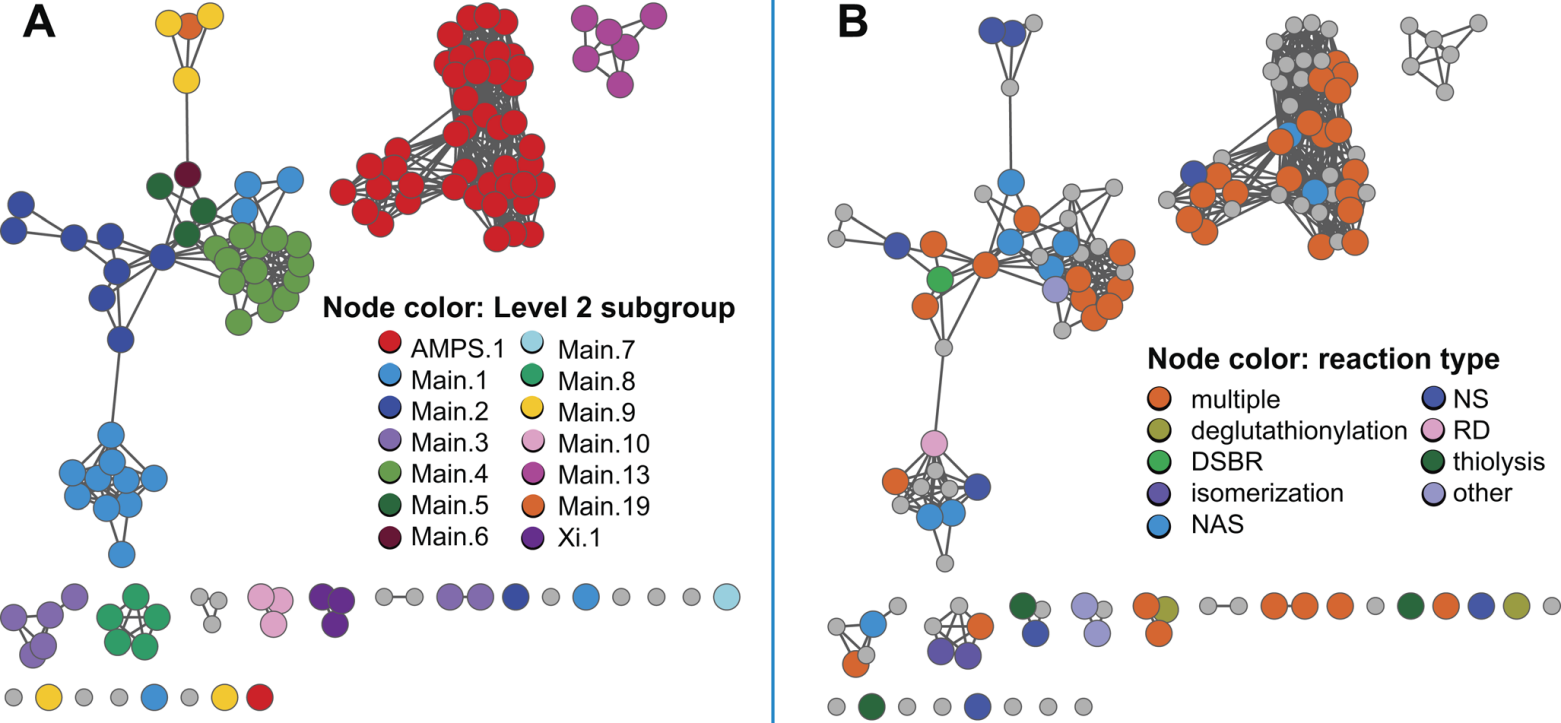
Representative structure similarity network for the cytGST superfamily. 131 representative structures for 379 cytGST structures filtered to 95% sequence identity are shown. Edges are shown as for Figure 1 except that structural similarity is defined from the FAST algorithm, with a FAST SN score ≥20 required to show edges. 565 edges are shown. For this network the median SN score is 22.7 over 187 residues. (A) Nodes are colored by level 2 subgroup assignments. (B) Nodes are colored by reaction type. As with the level 2 sequence similarity network, multiple reaction types are broadly spread throughout the structure similarity network, indicating that some divergent structures catalyze the same reaction types. Reaction type abbreviations: multiple, multiple reaction types present; DSBR, disulfide bond reductase; NAS, nucleophilic aromatic substitution; NS, nucleophilic substitution; RD, reductive dehalogenase.

Figure 5A shows a structure similarity network generated from all-by-all comparison of 131 representative structures and colored according to the largest level 2 subgroups. The cutoff threshold used for visualization of this network (FAST SN score ≥20) is highly statistically significant (see Methods) and was chosen to match roughly that used to visualize the level 1 sequence similarity network. As has been previously observed [27], the superfamily members share remarkable overall structure similarity despite the great diversity of their underlying sequences. Consistent with this observation, application of a more stringent threshold to the network shown in Figure 5A still fails to separate the nodes into well-resolved clusters.

The similarity of this structure-based network (Figure 5) to the sequence networks provides support for the clustering pattern shown in Figure 1 but also reveals how the Main level 2 subgroup structures are related to each other. We note that the structure similarity network generally, but not completely, corresponds to the sequence similarity relationships shown in Figures 1–3. For the AMPS.1 subgroup, the structures mostly group together, indicating they share much overall structural similarity. In contrast, the other major structure similarity cluster has mixed membership from diverse sequence similarity subgroups and some of these subgroups, such as Main.9, are split into more than one structure cluster, indicating their structures are quite divergent.

As with the sequence similarity network shown in Figure 3, the structure similarity network shown in Figure 5B, colored by reaction type, shows that reaction types generally do not cluster by subgroup or by annotated class (see Figure S2), but are represented broadly across many subgroups. However, some trends indicating structure similarity correlation with individual reaction types may be seen when the network is viewed painted with reaction types one at a time (unpublished data). For example, when nodes are highlighted only if there is evidence for dehydroascorbate reductase activity, it is observed that this reaction type occurs only in small outlier clusters well away from the largest two groupings. The structure similarity network available from the SFLD may be viewed by each individual reaction type as well as by the color coding scheme shown here in Figure 5B that indicates multiple or single reaction types. Though beyond the scope of this work, we note that because active sites may offer better discrimination of structural features associated with different reaction types, creation of structure-similarity networks based on similarity of conserved active site features rather than overall structure could reveal stronger correlation of structure with reaction type.

Although the structure similarity network offers a higher confidence determination of distant similarities among subgroups than can the sequence similarity networks, far fewer structures are available than are sequences. The latter include many thousands of unknowns that are missing from the structure similarity network. Thus, the structure similarity network is insufficient to describe the global relationships among the proteins of the superfamily. Rather, they complement the sequence similarity networks and reveal connections among level 2 subgroups that are not evident from the sequence data (Figure 2).

### Phylogenetic Representation of cytGSTs

**Figure 6:**
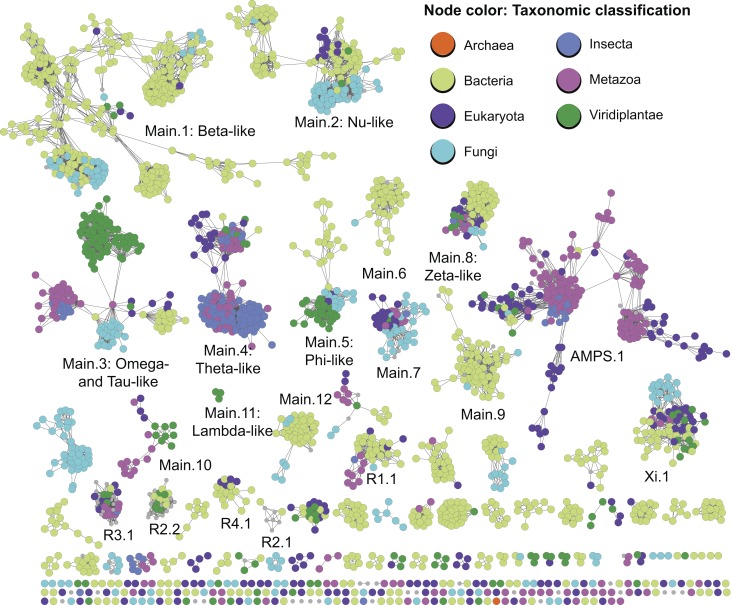
Level 2 sequence similarity network painted by type of life. The nodes of this level 2 representative network are colored by the type of life represented if more than 50% of the annotated member sequences in a representative node have that classification. Taxonomic classifications were labeled and ordered by the class Insecta, the kingdoms Metazoa, Viridiplantae, and Fungi, and the superkingdoms Eukaryota, Bacteria, and Archaea. Grey nodes indicate nodes in which there were not a majority of annotated nodes for one of these classifications or if annotations were not available from NCBI.

Figure 6 shows the level 2 subgroup network painted by taxonomic classification describing several “type of life” categories of general interest, including insect cytGSTs, discussed further below. Non-insect nodes were painted according to general categories describing type of life, and by kingdom and superkingdom if they did not belong to these categories. The cytGSTs were first discovered in mammals and cytGST studies initially focused more attention on eukaryotic and especially metazoan proteins [56]. However, previous studies [27] and the results reported here show that the great majority of cytGSTs discovered thus far come from eubacteria. As with reaction types, Figure 6 shows clearly that sequence-based subgrouping of these GSTs does not track well with phylogenetic representation. For example, although many of the metazoan cytGSTs that have been most well studied are found in the AMPS.1 subgroup, many are found in other subgroups as well. Conversely, a substantial number of AMPS.1 sequences are not from metazoans, but represent other eukaryotes, including insects, fungi, and green plants (Viridiplantae), and even include a small group of bacterial sequences. Interestingly, the cytGST superfamily even has a few (16) proteins from the superkingdom Archaea. These are halobacteria that occur mostly in subgroup Xi.1, although the archaeal coloring does not show up in this subgroup because in the view shown, non-archaeal designations of other member sequences dominate in the representative nodes. However, one outlier node of three halobacterial sequences with archaeal coloring can be seen near the bottom of Figure 6.

The disconnect between sequence similarity and phylogenetic representation is observed in the other subgroups of cytGSTs. These patterns, along with the broad promiscuity that is a signature feature of cytGSTs, make it difficult to classify uncharacterized superfamily members. The insect cytGSTs, for which historical classifications remain challenging to clarify, provide an example.

#### Challenges for classification and functional inference in insect cytGSTs

**Figure 7:**
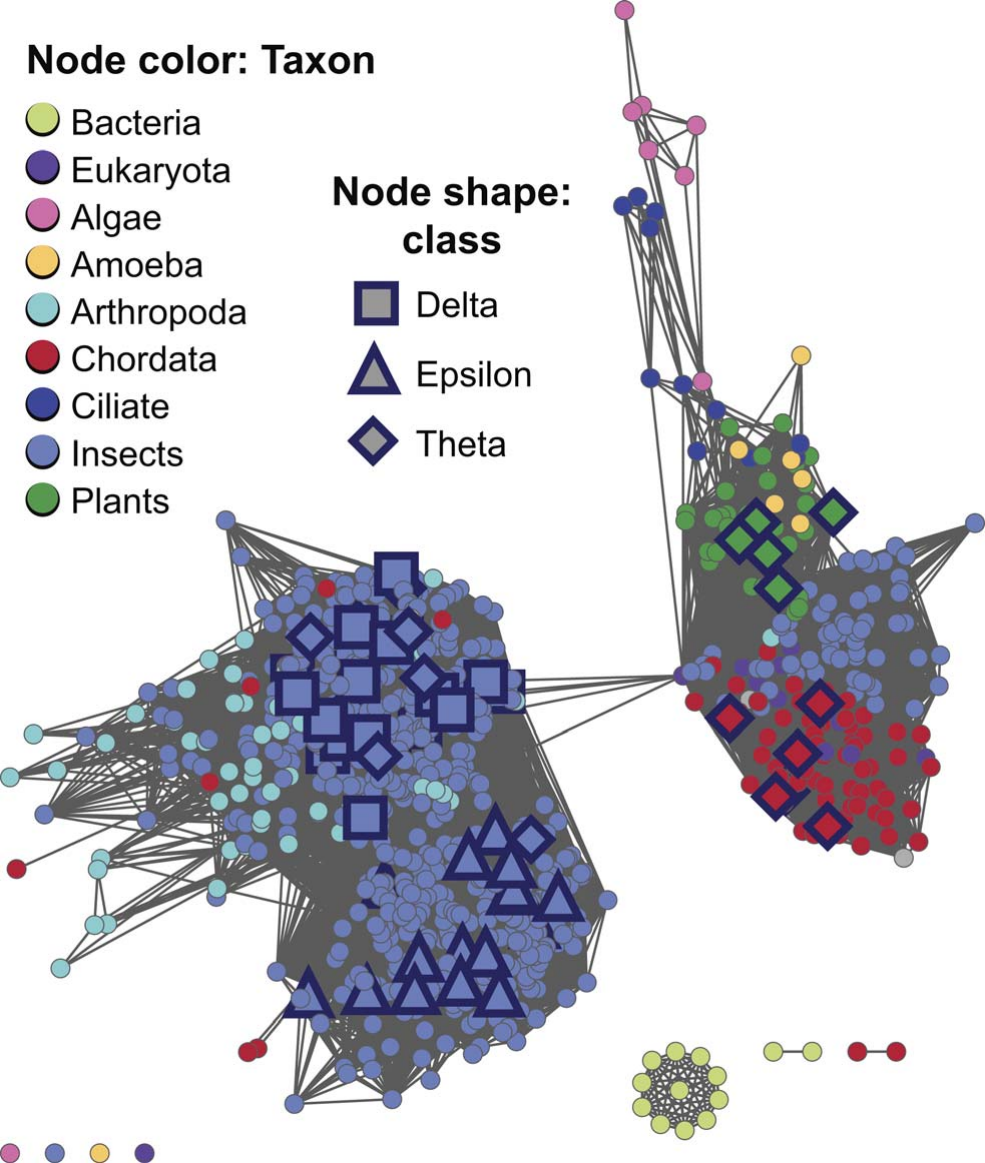
Full sequence similarity network of level 2 subgroup Main.4 indicating conflicting classifications from the literature. Edges are shown if they meet the similarity threshold of a BLAST *E*-value≤1×10^−31^. Shapes indicate class annotation from the literature: square, Delta; triangle, Epsilon; and diamond, Theta. To show more detail, colors are for taxonomic classifications from NCBI Taxonomy at a finer grained level than that used in the representative network shown in Figure 6. There are 872 sequences in the network with a median percent sequence identity for the 92,539 edges of 44% over 209 residues. Literature references for class annotations can be obtained from the network file for this subgroup available for download from the SFLD.

In addition to fundamental roles in metabolism and protection against oxidative stress, insect cytGSTs have been studied for many decades because of their role in resistance to insecticides, including organochlorine insecticides such as dichlorodiphenyltrichloroethane (DDT). Historically, insect cytGSTs were classified using several different nomenclature schemes. This has resulted in some confusion about their distribution across the canonical class designations. (See Ketterman [22] for a recent review of insect cytGSTs and their nomenclature.) For example, some *Drosophila* cytGSTs were originally misclassified into the Theta class, and later were re-classified as Delta class [22],[57]. The network view in Figure 6 shows that most insect cytGSTs (640 of the 916 insect sequences) occur in the subgroup designated as Main.4 (Theta-like) and represent the predominant type of life in this subgroup. Annotations for insect cytGST classes obtained from the literature show their distribution mapped onto the more detailed full network of Main.4 (Figure 7). These results show that the majority of characterized cytGSTs from insects have now been assigned to the Delta and Epsilon classes (including two re-classified *Drosophila* sequences specifically mentioned by Board et al. [57]). The designations Delta and Epsilon are not used by Swiss-Prot, adding to the challenges of sorting out these relationships from a global analysis.

The full network shown in Figure 7 is thresholded at an *E*-value cutoff of 1×10^−31^. At this threshold, clustering obtained by BLAST-based grouping and MCL correlated well (Text S1). In this view, most sequences that have been annotated by workers in the field as Theta class appear to form a natural cluster largely separated from the Delta and Epsilon groups. The main cluster shown on the left of Figure 7 also suggests that some inconsistencies still persist across the literature in the use of the Delta and Epsilon designations. The network indicates that some *bona fide* Theta class insect sequences may also exist (right cluster in Figure 7). As with the insect enzymes, the systematic application across the entire superfamily of the similarity criteria described by this work may be useful to experts in defining and correcting class boundaries for this and other subgroups as well. Misclassifications are not uncommon in this large and complex superfamily. For example, Sheehan et al. describe several other historical cases of misannotations/re-classifications involving plant and mouse cytGSTs [58].

While the representative network of Figure 6 shows insect cytGSTs to be most enriched in the Main.4 (Theta-like) subgroup, insect sequences are also found in other level 2 subgroups such as AMPS.1 and Main.3, but appear to be completely lacking in others such as subgroups Main.2, Main.5, and Xi.1. This is also consistent with the known patterns of broad and complex taxonomic distribution across most subgroups. The full network of Main.4 in Figure 7 also shows that while insects represent the majority of sequences, many non-insect arthropod, chordate, plant, and other cytGST sequences are quite similar to insect cytGSTs and are found together with them in this subgroup.

### DSBR Activity Is Broadly Represented across the Superfamily

As with taxonomic distribution, many reaction types are also represented in multiple subgroups of the cytGSTs. For example, a substantial number of divergent members of the cytGST superfamily have been experimentally determined to reduce low molecular weight disulfides using GSH (DSBR activity), a reaction that is also catalyzed by some members of the glutaredoxin superfamily [59],[60]. While most of the physiological substrates for this reaction remain to be identified, the presence of DSBR activity may be detected using any type of disulfide as a substrate provided that it readily forms a mixed disulfide with glutathione (Figures 4 and S3) [60],[61]. The most commonly used substrate is hydroxyethyl disulfide.

**Figure 8:**
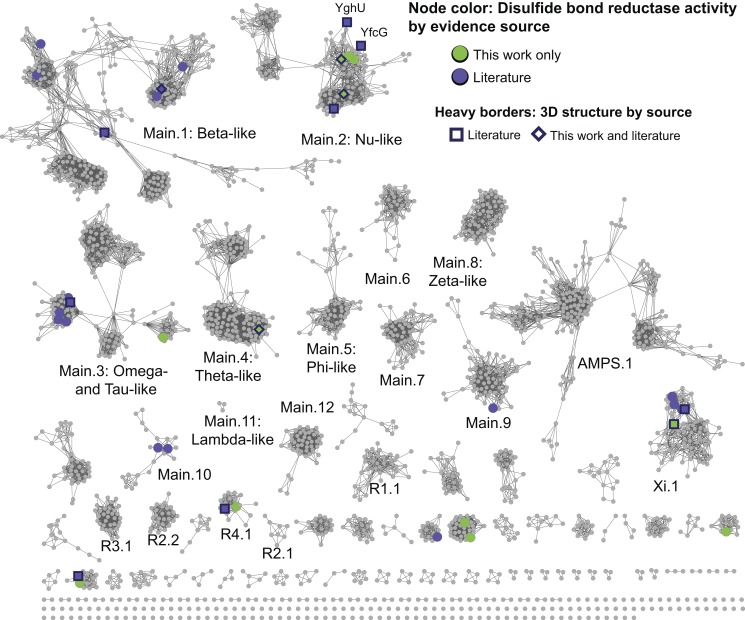
New experimental evidence for DSBR activity in many level 2 subgroups. The level 2 sequence similarity network is painted by DSBR activity. Nodes are colored if one or more member sequences in a representative node have experimental evidence for DSBR activity. Green indicates the evidence comes from only from this work and purple indicates evidence is from the literature. One representative node, labeled with member sequence YghU, has evidence both from the literature and this work. Heavy borders indicate that a 3D crystal structure is associated with at least one member sequence of a representative node where DSBR activity occurs and shape indicates the source of the structure data: square, from the literature; diamond, structure evidence both from this work and the literature.

**Figure 9:**
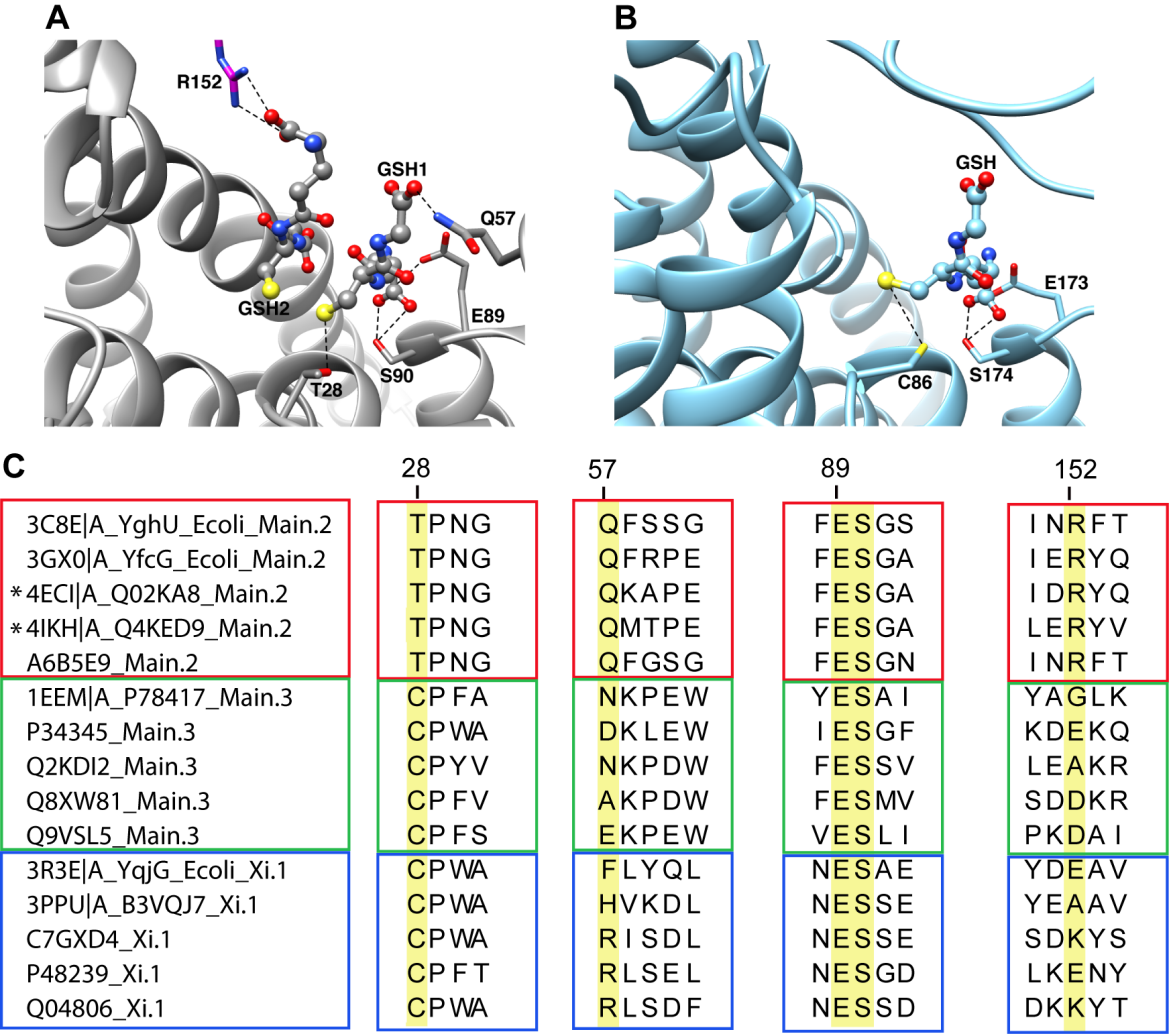
Comparison of the active site region from divergent subgroups with DSBR activity reveals some commonalities. (A) Structure of Q4KED9 (UniProt accession) from *Pseudomonas fluorescens* (PDB ID 4IKH, subgroup Main.2), one of the new structures from this work, showing two molecules of glutathione bound in the active site. The interactions between the bound ligands and the side chains of Thr28, Gln57, Glu89, Ser90, and Arg152 of the other subunit (magenta) are shown. (B) Structure of B3VQJ7 from *Phanerochaete chrysosporium* (PDB ID 3PPU, subgroup Xi.1) with one molecule of glutathione bound. The corresponding interactions between glutathione and the side chains of Cys86, Glu173, and Ser174 are shown. (C) Summary of sequence motifs from the structure-guided alignment showing the sequence context for the residues highlighted in 7A and 7B from several divergent subgroups: Main.2 (red box), Main.3 (green box), and Xi.1 (blue box). All the proteins shown have experimental evidence for DSBR activity. UniProt entries and available PDB IDs are given on the left side of the sequences. Highlighted in yellow are the aligned positions of the residues that have notable interactions with the bound ligand as described in the text (numbered according to 4IKH/Q4KED9). New structures generated for this work are indicated with an asterisk.

The distribution of proteins across the cytGSTs confirmed to catalyze the DSBR reaction is provided in Figure 8, showing that this reaction type is widely distributed throughout the superfamily. There are 44 sequences in 36 representative nodes that have experimental evidence for this activity (16 determined by this work and 28 proteins reported in the literature); 13 of these proteins have been structurally characterized [24],[25],[62]–[67] including three proteins with structures determined for this work (PDB codes 4ECI and 4ECJ [UniProt Q02KA8], 4HI7 [B4KM86], and 4IKH [Q4KED9]). The Main.2 and Main.3 subgroups contain the largest number of sequences that exhibit DSBR activity. Figure 9A and 9B show active site residue interactions with the substrate(s) from two of the most remotely related subgroups that include proteins with DSBR activity: Main.2 (using a structure from this work, 4IKH from *Pseudomonas fluorescens*, as the example structure in Figure 9A) and Xi.1 (using 3PPU from *Phanerochaete chrysosporium* as the example structure in Figure 9B). Sequences in these two subgroups share only 14% sequence identity (mean pairwise %ID calculated from the alignment). Figure 9C provides a view of the sequence context for the residues labeled in Figure 9A and 9B. These motifs, generated from a structure-guided MSA of 44 sequences with experimentally confirmed DSBR activity from 11 level 2 subgroups, summarize key similarities and differences among additional divergent proteins with DSBR activity. The full MSA that includes all 44 sequences is provided in Figure S4.

#### Natural variations in the machinery required for DSBR catalysis across the superfamily

Structural and functional studies on Main.2 (Nu-like) subgroup members YfcG [64] and YghU [24] from *E. coli* showed binding in the active site to either two molecules of GSH or one molecule of oxidized glutathione (GSSG), respectively. We compared these proteins with other Main.2 structures and sequences with experimental evidence for DSBR activity, and found strong conservation of several active site residues likely to be important for binding the substrate (using 4IKH numbering): Thr28, Gln57, Glu89, Ser90, and Arg152 (Figure 9C). Thr28 forms a hydrogen bond to the sulfhydryl group of a bound glutathione, presumably facilitating the stabilization of the glutathione thiolate that acts as the nucleophile in the catalyzed reaction (Figure 9A). The utilization of a threonine residue in GST-catalyzed reactions has been previously observed [24],[64], but is unusual within the superfamily. Other members typically use cysteine, serine, or tyrosine residues in catalysis [2],[27]. An arginine residue (Arg152) in the second subunit of the enzyme interacts with the presumed second bound ligand. Also shown in Figure 9A are interactions that occur between various functional groups of bound GSH or GSSG with Gln57, Glu89, and Ser90. The strong conservation of these five highlighted residues in subgroup Main.2 proteins is shown in Figure 9C. These residues may thus represent a general motif that predicts Main.2 subgroup characteristics that include the binding of a second glutathione in the active site.

As illustrated in Figures 9C and S4, further comparison of all sequences and structures with evidence for DSBR activity across all the subgroups reveals the sulfhydryl-interacting residue at position 28 (using 4IKH numbering) is a Thr in Main.2 subgroup members but is typically a Cys and sometimes a Ser in other subgroups containing members with evidence for DSBR activity. This illustrates the variability nature has allowed for the role of this residue in DSBR activity. Across the superfamily, without regard to DSBR activity, this critical residue is most commonly a Tyr in the Alpha, Mu, Pi, and Sigma classes, and a Cys or Ser in enzymes in the level 1 Main subgroup [2],[27].

Inspection of the available structures from non-Main.2 subgroups with DSBR activity shows that there is a lack of conservation of residues corresponding to Main.2 Arg152 and Gln57 (4IKH numbering) and inspection of the MSA shows that no clear alternative sub-motif exists (Figures 9C and S4). Additional investigation will be needed to determine if alternative residues in non-Main.2 subgroup members fulfill similar roles to Main.2 Arg152 and Gln57 in interacting with bound ligand in DSBR reactions. In contrast to Arg152 and Gln57, the dipeptide of Glu89 and Ser90 is highly conserved across all subgroups with DSBR activity, though in a few cases, the Glu is substituted with an Asp (Figure S4). It is known that these two residues occur in a core ββα region that is highly conserved across many divergent cytGSTs and have been described as a Gln or Glu residue followed by a Ser or Thr among Alpha, Mu, Pi, Sigma, and Theta class members [2]. The strongly conserved ES dipeptide in the proteins depicted in Figure 9 reveals an important commonality that perhaps suggests a shared evolutionary ancestor linking proteins from the extremely divergent level 2 Main and Xi subgroups.

### cytGSTs Catalyze Unusual Reactions: Bioremediation of Environmental Pollutants

**Figure 10:**
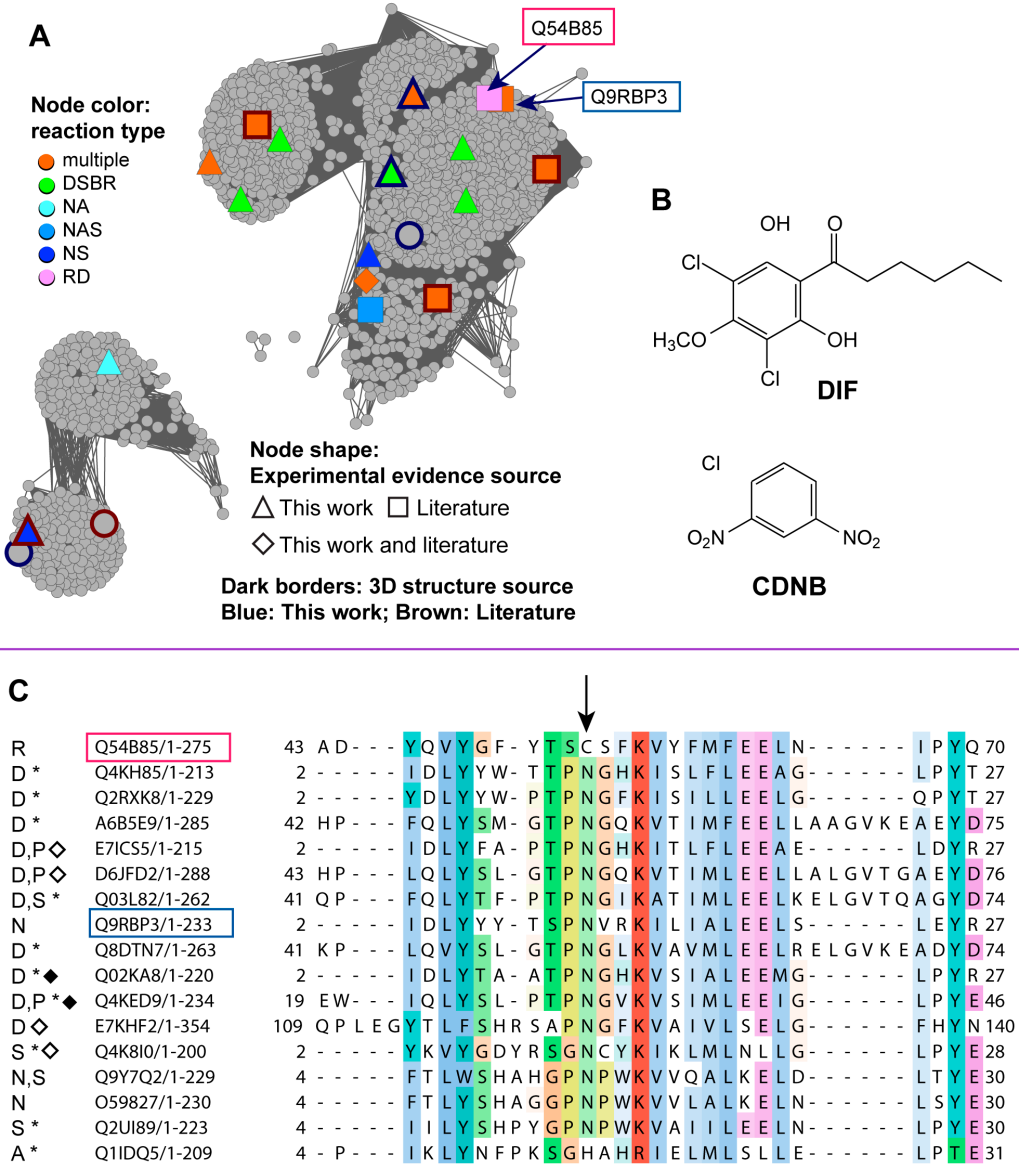
Similarity relationships between a reductive dehalogenase protein and some homologs may give insight into function. (A) The full sequence similarity network view of Main.2 displays all 1,642 non-redundant sequences for this level 2 subgroup. Boxed labels and arrows indicate the slime mold protein with reductive dehalogenase activity (Q54B85) and its closest homolog Q9RBP3. Nodes are colored if there is experimental evidence for cytGST-like function with shapes indicating the evidence source: triangle, this work; square, the literature; diamond, both this work and the literature. Dark borders indicate nodes with crystal structures; the border color indicates the source of the structure: blue, this work; brown, the literature. Node colors designate the reaction type(s) associated with each sequence (node): multiple, multiple reaction types; DSBR, disulfide bond reductase; NA, nucleophilic addition; NAS, nucleophilic aromatic substitution; NS, nucleophilic substitution; RD, reductive dehalogenase. Edges with BLAST *E*-values≤1e–31 are shown; the 233,255 edges shown in the network have a median percent ID of 54% over 214 residues. (B) Chemical structures of DIF that is reductively dehalogenated by Q54B85, and CDNB, the synthetic compound commonly used in NAS assays. (C) Alignment of Q54B85 (red box) with homologs that have experimental evidence for GST-like activity. An arrow indicates residue Cys54 from Q54B85 that is critical for RD activity but is a conserved Asn in most homologs. The blue box indicates Q9RBP3, the closest homolog of Q54B85 that has CDNB activity. Reactions designated in the alignment are as follows: R, RD; D, DSBR; N, NAS; S, NS; A, NA; P, peroxidase. Sequences marked with an asterisk indicate that the experimental evidence for that reaction was obtained only from this work; sequences marked with a diamond indicate the availability of a crystal structure for that protein and black diamonds indicate crystal structures are from this work.

An examination of the full network of the Main.2 (Nu-like) subgroup (Figure 10A) shows many unknowns, with only a few members that have been biochemically and structurally characterized. One of these, the cytGST-like protein Q54B85 (DrcA), is a eukaryotic reductive dechlorinase (reductive dehalogenation reaction type) (Figure 4) for which a natural substrate, differentiation-inducing factor (DIF), is known [68]. DIF is a chlorinated alkyl phenone found in *Dictyostelium discoideum* (slime mold) and induces stalk cell differentiation under starvation conditions. The chemical structures of DIF and the commonly used cytGST synthetic substrate CDNB are shown in Figure 10B. How dehalogenation of organic compounds occurs in nature is of interest because of the importance of these reactions for biodegradation of synthetic organohalides such as DDT, dioxins, and polychlorinated biphenyls (PCBs) that persist in the environment as toxic pollutants. Understanding how enzymes have evolved to dehalogenate naturally halogenated compounds in an efficient way may provide guidance for engineering enzymes in the lab for bioremediation and other specialized chemistry.

In an experiment that addresses this challenge, Velazquez and colleagues [68] showed that Cys54 of DrcA is critical for reductive dechlorinase activity and that in C54N mutants, the step in which DIF is conjugated by GSH is left intact but that the second step that generates free GSH and the reduced dechlorinated substrate does not occur. This suggests that Cys54 is required for full reductive dechlorinase activity. As indicated in Figure 10C, alignment of DrcA with homologs from Main.2 for which experimental evidence confirms cytGST-like activity shows that most have an Asn residue in the position that corresponds to Cys54 in DrcA. There are a total of 17 Main.2 sequences with experimentally confirmed GST-like activity, and of these, ten have experimental evidence only from this work. Two of these homologs have been shown to catalyze GST chemistry using CDNB as a substrate, but some others do not, even though they possess an Asn in this position. It is an open question whether mutating the conserved Asn to a Cys (perhaps along with other mutations) could confer reductive dechlorination activity to those cytGST homologs that exhibit CDNB activity, especially in the sequence in Main.2 most closely related to DrcA, Q9RBP3. A number of other cytGSTs have been reported to play roles in detoxification of toxic pollutants and are under investigation for bioremediation efforts. One well-known example is a pathway found in *Sphingomonas* that degrades pentachlorophenol in which two cytGST enzymes play roles [69]–[71].

### Conclusions

As sequence databases continue to grow much faster than the encoded proteins can be experimentally characterized, new approaches are required to infer structure-function relationships among the majority of proteins of unknown function. Large-scale analysis of what is known and unknown across the entirety of large superfamilies of homologous proteins offers one strategy for leveraging experimental information to address this challenge. Here, we have described relationships across the members of the cytGST superfamily from a global context defined by sequence similarity. To aid in visualization and interpretation of the results, we used sequence similarity networks to illustrate these relationships at several levels of detail and mapped to them known classifications, structures, reaction classes, and the distribution of these proteins across the biosphere. We neither suggest that the subgroupings that result and the subclusterings shown within them are replacements for the canonical classes nor that they rigidly define new classes; rather, they could provide a foundation for an expanded classification of cytGSTs to include the majority of unclassified unknowns. They may also help to resolve inconsistencies in the canonical classifications of knowns arising from the ad hoc manner in which they were historically assigned. Additionally, the new network views provided here show how the use of the large-scale data now available reveals patterns that relate sequence and structure to function in ways that would be difficult to obtain from smaller-scale approaches.

The new experimental results reported here expand substantially the number of unknowns that can now be confirmed to catalyze a GST-like reaction. Further, they expand structural coverage into several highly divergent clusters that previously had little structural representation. Using DSBRs as an example, this work also illustrates from a global perspective the substantial overlap of this reaction type across the many subgroups of the superfamily. As shown in Figure 4, this theme of promiscuity and widely overlapping reaction types across many subgroups provides new insight about why inference of their functions is an especially difficult task. Likewise, the global view of taxonomic representation across the superfamily suggests a complex pattern that does not track well with subgroupings by sequence or structure similarity, complicating further our understanding of the evolution of the cytGSTs.

While the results from this study provide new insight for a global understanding of structure-function relationships for the cytGST superfamily, many challenges still remain, among them the difficulty in obtaining functional information on the scale that is available from sequence data. Further, unlike sequence and structure data that are deposited by standard practice into public databases, much experimental data about proteins are not deposited systematically into centralized, cross-referenced databases annotated with specific accession identifiers, so that even obtaining all available functional data for systematic analysis of an entire superfamily remains challenging.

## Supporting Information

Figure S1 **Distribution of cytGST lengths in the SFLD.** The lengths of all cytGST proteins in this study were binned by length (full-length sequences were used).
(PDF)

Figure S2 **Structure similarity network colored by canonical class.** The network is the same as that shown in Figure 5, except nodes are colored according to canonical cytGST classes from Swiss-Prot annotations, and by the Xi and Nu classes. Sequences from the Alpha, Mu, Pi, and Sigma classes share similar overall structures, consistent with the sequence similarity network shown in Figure 2. In contrast, proteins from other classes group together in the other major structure cluster shown in the figure, despite their quite divergent underlying sequences.
(EPS)

Figure S3 **Examples of some experimentally known disulfide substrates for cytGSTs with DSBR activity.**
(EPS)

Figure S4 **Full structure-guided MSA from which the summary motif alignment shown in** **Figure 9C** **was created.** PDB IDs and UniProt entries for sequences without structures are given on the left of the alignment. Sequences for proteins with DSBR activity were used in the alignment, some of which also had structures as indicated. In order to help guide the alignment for subgroups that lacked structures with evidence for DSBR activity, two available structures from these subgroups were included in the alignment (indicated by “NO_DSBR” in the labels). Higher conservation is indicated by more intense colors. The five highly conserved positions in the Main.2 (Nu-like) subgroup discussed in the main text are indicated with arrows, and subgroups represented in Figure 9C are boxed and color-coded as in the legend for Figure 9. The strongly conserved ES (Glu-Ser) pair that is noted in the text for the summary alignment is also shown to be highly conserved in the full alignment shown; although for a few sequences an Asp is substituted for Glu.
(PDF)

Table S1 **High throughput assays used in this work.** The assays for cytGST-like activity used in this work are shown, categorized by reaction type, and the substrates used are indicated. References for the methods are also given.
(DOCX)

Table S2 **Total counts of representative nodes and member sequences in level 1 and level 2 subgroups.**
(XLSX)

Table S3 **Experimentally verified cytGST reaction types for each representative nodes in networks provided in this work.** “Multiple reaction types in node” means that there is evidence for more than one reaction type in one or more member sequences in a representative node.
(XLSX)

Table S4 **Experimentally characterized cytGSTs and their substrates.** Proteins with experimental confirmation of cytGST-like activity are listed. “This work” means the experimental data are from assays run for this work. Column titles and definitions: accn, UniProt accession; ID in network, identifier for a non-redundant protein used in the full networks; reference, PubMed ID or “This work”; source, database from which evidence was extracted or “This work”; efdid, protein identifier for accessing a protein in the SFLD; link to protein in the SFLD, URL address to the protein in the SFLD. The full names for shortened level 2 subgroup names in the table are: AMPS.1: Alpha-, Mu-, Pi-, and Sigma-like; Main.1: Beta-like; Main.2: Nu-like; Main.3: Omega- and Tau-like; Main.4: Theta-like; Main.5: Phi-like; and Main.8: Zeta-like.
(XLSX)

Table S5 **3D structures for cytGSTs.** The 3D structures mapped onto the networks in this work are listed; new structures from this work were solved by the EFI, and are indicated by the presence of an EFI target ID. Primary ID designates the protein ID used in this paper (usually UniProt accession number, or an EFI target ID [see Methods]) to represent redundant protein sequences. The Primary ID may differ from the dbAccession, which is the ID in the PDB file that lists the target ID for the cognate sequence for that structure. If a dbAccession in the PDB file was not a UniProt identifier, a UniProt identifier is provided in this Table (UniProt column). The column “GST activity” indicates the source of the experimental data if there is evidence for GST-like activity: “This work” means the experimental data were obtained only from assays provided in this work; “Lit” means the evidence comes from the literature; “This work and Lit” means the evidence comes from both sources.
(XLSX)

Text S1 **How thresholds for viewing networks were chosen.**
(PDF)

## Abbreviations

CDNB: 1-chloro-2,4-dinitrobenzene
cytGST: cytosolic glutathione transferase
DIF: differentiation-inducing factor
DSBR: disulfide bond reductase
EFI: Enzyme Function Initiative
GSH: glutathione
GST: glutathione transferase
PDB: Protein Data Bank
SFLD: Structure-Function Linkage Database
