# Supplementary figures and images for: Large-Scale Determination of Sequence, Structure, and Function Relationships in Cytosolic Glutathione Transferases across the Biosphere
Source: PLoS Biol. 2014 Apr 22;12(4):e1001843. doi: 10.1371/journal.pbio.1001843 (PMC3995644; doi:10.1371/journal.pbio.1001843)

## Distribution of cytGST lengths

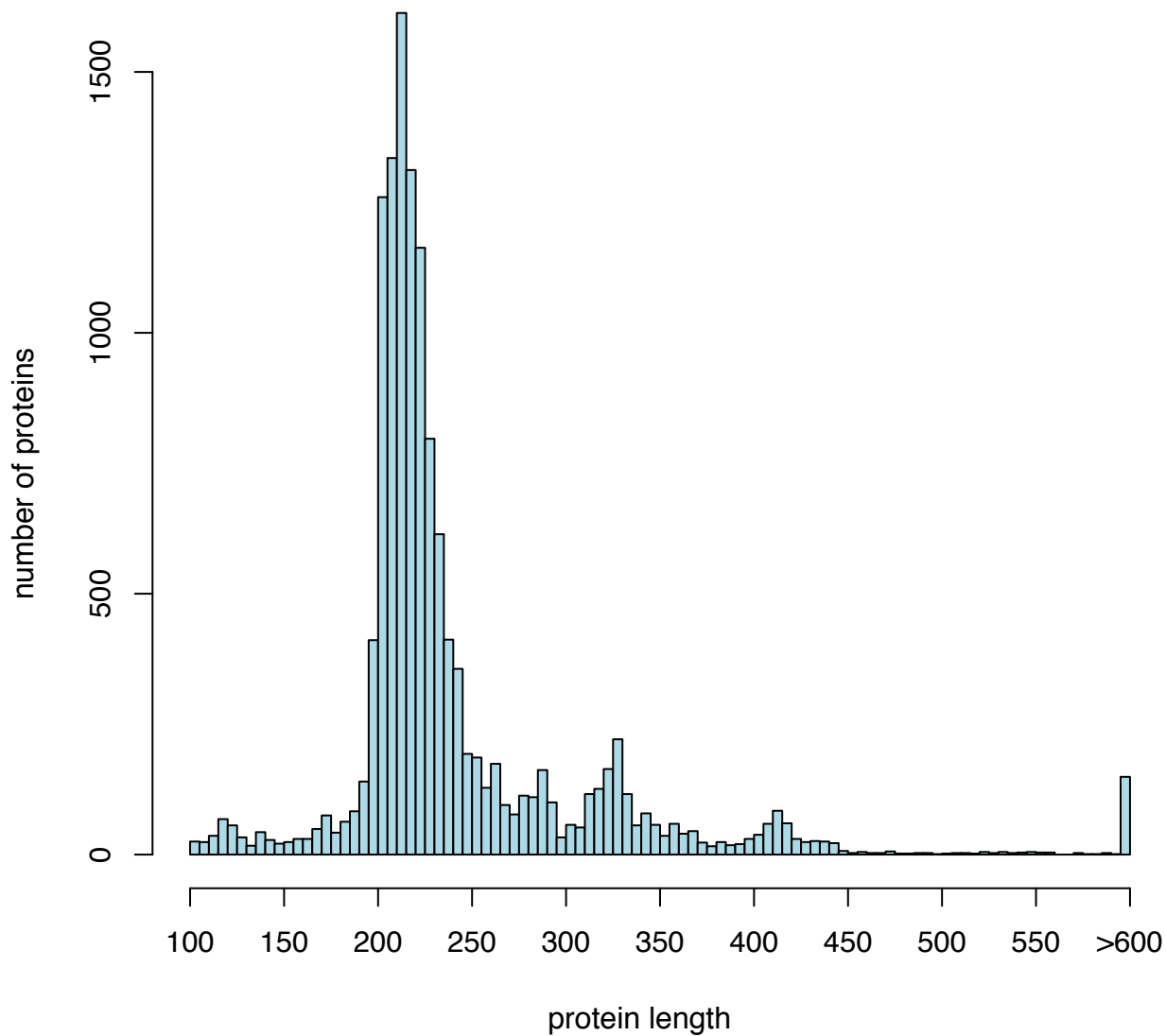

Supplement: Figure S1 — Distribution of cytGST lengths in the SFLD. The lengths of all cytGST proteins in this study were binned by length (full-length sequences were used). (PDF) [file pbio.1001843.s001.pdf]
