# Supplementary material for: Large-Scale Determination of Sequence, Structure, and Function Relationships in Cytosolic Glutathione Transferases across the Biosphere
Source: PLoS Biol. 2014 Apr 22;12(4):e1001843. doi: 10.1371/journal.pbio.1001843 (PMC3995644; doi:10.1371/journal.pbio.1001843)
Supplement: Figure S4 — Full structure-guided MSA from which the summary motif alignment shown in Figure 9C was created. PDB IDs and UniProt entries for sequences without structures are given on the left of the alignment. Sequences for proteins with DSBR activity were used in the alignment, some of which also had structures as indicated. In order to help guide the alignment for subgroups that lacked structures with evidence for DSBR activity, two available structures from these subgroups were included in the alignment (indicated by “NO_DSBR” in the labels). Higher conservation is indicated by more intense colors. The five highly conserved positions in the Main.2 (Nu-like) subgroup discussed in the main text are indicated with arrows, and subgroups represented in Figure 9C are boxed and color-coded as in the legend for Figure 9. The strongly conserved ES (Glu-Ser) pair that is noted in the text for the summary alignment is also shown to be highly conserved in the full alignment shown; although for a few sequences an Asp is substituted for Glu. (PDF) [file pbio.1001843.s004.pdf]

1A0F|A_E2KU21_Main.1/1-201	1	---MK	2
A9CII7_Main.1/1-208	1	--MSELI	5
A9CJU4_Main.1/1-219	1	--MLT	3
A9CJU5_Main.1/1-206	1	--MFT	3
A9CLG1_Main.1/1-203	1	--MLT	3
Q7D3T0_Main.1/1-211	1	---MK	2
3C8E|A_YghU_D6JFD2_Main.2/1-284	1	---TYQPAKVWTWDKSAGGAFANI---NRPVS----GPTHE-----------------KTLP-VGK-HPLQ	42
3GX0|A_YfcG_E7ICS5_Main.2/1-204	1	--MID	3
1G6W|A_E7KHF2_Main.2/1-234	1	--------------------------------------SRI-----------------TKFFQEQPLEGYT	16
4ECI|A_Q02KA8_Main.2/1-204	1	---VMID	4
4IKH|A_Q4KED9_Main.2/1-227	1	--------------------SAF---------------AVT-----------------QKWP-AQFPEWIQ	18
Q03L82_Main.2/1-262	1	-MSDYILPEVWENPTSMGGTWGGL---NQPTA----GARFE-----------------QTLP-KGD-QPFQ	44
Q8DTN7_Main.2/1-263	1	-MSYYIPPKVWSAEESNQGKFSAI---NRPTA----GSRFD-----------------QKLP-QGD-KPLQ	44
A6B5E9_Main.2/1-285	1	MANEYTPPRVWTNDSENGGQWASI---NRPDS----GARHE-----------------QELP-VGE-HPFQ	45
Q2RXK8_Main.2/1-229	1	--MYD	3
Q4KH85_Main.2/1-213	1	--MID	3
1EEM|A_P78417_Main.3/1-237	1	---------------------------SARSL-----GKGS-----------------APPG-PVPEGSIR	21
P34345_Main.3/1-250	1	------------------MVLTGV---TSKAI-----RKGD-----------------AEP--PLSKGSFR	26
Q2KDI2_Main.3/1-227	1	--MSRLT	5
Q8XW81_Main.3/1-224	1	--MPDSTLT	7
Q9H4Y5_Main.3/1-243	1	--------------------MSGD---ATRTL-----GKGS-----------------QPPG-PVPEGLIR	25
Q9VSL2_Main.3/1-241	1	-------------------------MSSGKHL-----AKGS-----------------PKPV-LPDDGVLR	23
Q9VSL3_Main.3/1-243	1	-------------------------MSNGRHL-----AKGS-----------------PMPD-VPEDGILR	23
Q9VSL4_Main.3/1-250	1	---------------------MAL---PQKHF-----KRGS-----------------PKPE-IPEDGVLR	24
Q9VSL5_Main.3/1-251	1	---------------------MAL---PQKHF-----KRGS-----------------TKPE-LPEDGVPR	24
Q9VSL6_Main.3/1-254	1	-------------------------MSNTQHL-----TIGS-----------------PKPV-FPDDGILK	23
4HI7|A_B4KM86_Main.4/1-220	1	--KPI	3
3R2Q|A_B3HZI4_Main.9_NO_DSBR/1-202	1	---MK	2
A9CJG4_Main.9/1-198	1	---ME	2
2R4V|A_O15247_Main.10_NO_DSBR/1-226	1	---VDPEIE	6
Q8LE52_Main.10/1-258	1	-------------------MISLRFQPSTTAGVLSASVSRAGFIKRCGSTKPGRVGRFVTMA-TAAS-PLE	50
Q9FRL8_Main.10/1-213	1	---MALD	4
Q9FWR4_Main.10/1-213	1	---MALE	4
Q88LB2_Main.14/1-199	1	---MRVI	4
Q129E0_Main.14/1-230	1	-------------------------------------MTPA-----------------STAP--ADRPLPV	15
A9CKF2_Main.15/1-230	1	--MPT	3
2YCD|A_A9CFJ9_Main.25/1-213	1	--KPNPTIT	7
B8H2E2_Main.25/1-217	1	---MTPVIS	6
B7UV05_Main/1-220	1	---MSLT	4
1G7O|A_B3BP81_R4.1/1-215	1	---MK	2
D0W558_R4.1/1-213	1	---MK	2
3PPU|A_YqjG_B3VQJ7_Xi.1/1-314	1	---------------ELQSDISKM---KTEDDG-SFKRKAASFRN--------WIQPNGDFT--PEKGRYH	42
3R3E|A_C4ZR18_Xi.1/1-308	1	-------------------GQLID---------GVWHAFRNWLTADG----APGPTGTGGFI--AEKDRYH	37
C7GXD4_Xi.1/1-370	1	-------------------MSKQW---ASGTNG-AFKRQVSSFRET-------ISKQHPIYK--PAKGRYW	39
P48239_Xi.1/1-356	1	----------------------------MSVSYKGTISKTH-----------------SVFK--PEKGRYY	24
Q04806_Xi.1/1-366	1	-------------------MSEKS---ASNNKA-EFKRQSSPFREI-------ISADHPIYK--PAKGRYW	39

Sequence	Start	Alignment	End
1A0F|A_E2KU21_Main.1/1-201	3	LF---------YKP---------GACSLASHITLRES------GKD--FTLVSV--DLMKKR---------	36
A9CII7_Main.1/1-208	6	FY----------TNP--------MSRGRIARWMLEEV------GIP--YKTEIL---GFETS---------	38
A9CJU4_Main.1/1-219	4	IY---------GVYR---------SRATRTLWLAAEL------GIE--FKHVPV--IQARRLADPLA----	42
A9CJU5_Main.1/1-206	4	LF---------FSP---------GSCSRASHIVLEES------GLP--YKAHRV--NFAEGE---------	37
A9CLG1_Main.1/1-203	4	LF---------HAPR---------SRSTRIVTLLRELD----AIDK--VAIKIV--DITRGD---------	39
Q7D3T0_Main.1/1-211	3	LY---------YMP---------AACSLSPHIVANEL------ELD--IEFVRV--NFKDHK---------	36
3C8E|A_YghU_D6JFD2_Main.2/1-284	43	LY----------SLG--------TPNGQKVTIMLEELLALGVTGAE--YDAWLI--RIGDGD---------	82
3GX0|A_YfcG_E7ICS5_Main.2/1-204	4	LY----------FAP--------TPNGHKITLFLEEA------ELD--YRLIKV--DLGKGG---------	37
1G6W|A_E7KHF2_Main.2/1-234	17	LF---------SHRS--------APNGFKVAIVLSEL------GFH--YNTIFL--DFNLGE---------	51
4ECI|A_Q02KA8_Main.2/1-204	5	LY----------TAA--------TPNGHKVSIALEEM------GLP--YRVHAL--SFDKKE---------	38
4IKH|A_Q4KED9_Main.2/1-227	19	LY----------SLP--------TPNGVKVSIMLEEI------GLP--YEAHRV--SFETQD---------	52
Q03L82_Main.2/1-262	45	LY----------TFP--------TPNGIKATIMLEELKELGVTQAG--YDAYRI--KIGDGD---------	84
Q8DTN7_Main.2/1-263	45	VY----------SLG--------TPNGLKVAVMLEELRELGVKEAD--YDLFKI--SIMDGD---------	84
A6B5E9_Main.2/1-285	46	LY----------SMG--------TPNGQKVTIMFEELLAAGVKEAE--YDAYLI--KIGDGD---------	85
Q2RXK8_Main.2/1-229	4	LY----------YWP--------TPNGFKISILLEEL------GQP--YTLKPV--NIAKGE---------	37
Q4KH85_Main.2/1-213	4	LY----------YWT--------TPNGHKISLFLEEA------GLP--YTLYPV--NIGLGE---------	37
1EEM|A_P78417_Main.3/1-237	22	IY---------SMRF--------CPFAERTRLVLKAK------GIR--HEVINI--NLK------------	53
P34345_Main.3/1-250	27	VY---------NMRF--------CPWAERAMLYVAAK------GIE--AEVVNL--NVT------------	58
Q2KDI2_Main.3/1-227	6	LI---------SHHL--------CPYVQRAAIALREK------GVP--FERINI--DLA------------	37
Q8XW81_Main.3/1-224	8	LI---------SHPL--------CPFVQRAAIVLLEK------GVP--FERINV--DLA------------	39
Q9H4Y5_Main.3/1-243	26	IY---------SMRF--------CPYSHRTRLVLKAK------DIR--HEVVNI--NLR------------	57
Q9VSL2_Main.3/1-241	24	LY---------SMRF--------CPYAQRAHLVLNAK------NVP--YHSVYI--NLT------------	55
Q9VSL3_Main.3/1-243	24	LY---------SMRF--------CPFAQRVHLVLDAK------QIP--YHSIYI--NLT------------	55
Q9VSL4_Main.3/1-250	25	YY---------SMRF--------CPYSQRAGLVLAAK------KIP--HHTVYI--DLS------------	56
Q9VSL5_Main.3/1-251	25	FF---------SMAF--------CPFSHRVRLMLAAK------HIE--HHKIYV--DLI------------	56
Q9VSL6_Main.3/1-254	24	LY---------SMRF--------CPYAHRVHLVLDAK------KIP--YHAIYI--NLR------------	55
4HI7|A_B4KM86_Main.4/1-220	4	LY---------GIDA--------SPPVRAVKLTLAAL------QLP--YDYKIV--NLMNKE---------	38
3R2Q|A_B3HZI4_Main.9_NO_DSBR/1-202	3	LV---------GSYT--------SPFVRKLSILLLEK------GIT--FEFINE--LPY------------	34
A9CJG4_Main.9/1-198	3	LL---------YSPA--------SPYSAKVRMAARHL------DID----VTSV--RVDTN----------	34
2R4V|A_O15247_Main.10_NO_DSBR/1-226	7	LF---------VKAGSDGESIGNCPFCQRLFMILWLK------GVK--FNVTTV-----------------	43
Q8LE52_Main.10/1-258	51	ICVKASITTPNKLGD--------CPFCQKVLLTMEEK------NVP--YDMKMV--DLS------------	91
Q9FRL8_Main.10/1-213	5	ICVKVAVGAPDVLGD--------CPFSQRVLLTLEEK------KLP--YKTHLI--NVS------------	45
Q9FWR4_Main.10/1-213	5	ICVKAAVGAPDHLGD--------CPFSQRALLTLEEK------SLT--YKIHLI--NLS------------	45
Q88LB2_Main.14/1-199	5	LY---------SFRR--------CPWAMRARLALRYA------GCE----VEIC--EVAMK----------	36
Q129E0_Main.14/1-230	16	LY---------SFRR--------CPYAMRARLALVVS------GQR----CELR--EVVLR----------	47
A9CKF2_Main.15/1-230	4	LY---------HSPM--------STASRFVRLILAEY------GFQ--TDLVEE--QPW------------	35
2YCD|A_A9CFJ9_Main.25/1-213	8	VFER----SPDGGRG--------LARDMPVRWALEEV------GQP--YHVRRL----SFEA---------	45
B8H2E2_Main.25/1-217	7	AYNW----VPPPARG--------LVRDLRIRWALEEL------GRP--YAVETV--LH-------------	42
B7UV05_Main/1-220	5	LY---------GAPL--------SPFVRKVRLLLAEK------GLD--YQLEAI--APF------------	36
1G7O|A_B3BP81_R4.1/1-215	3	LY---------IYDH--------CPYCLKARMIFGLK------NIP--VELHVL----LND----------	34
D0W558_R4.1/1-213	3	LY---------IYDH--------CPFCVRARMAAGLF------GAD--VEEVVM----AND----------	34
3PPU|A_YqjG_B3VQJ7_Xi.1/1-314	43	LY---------VSYA--------CPWATRTLIVRKLK------GLEDFIGVTVVSPRMGSNGWPFANVDPF	90
3R3E|A_C4ZR18_Xi.1/1-308	38	LY---------VSLA--------CPWAHRTLIMRKLK------GLEPFISVSVVNPLMLENGWTFDDSFPG	85
C7GXD4_Xi.1/1-370	40	LY---------VSLA--------CPWAHRTLITRALK------GLTSVIGCSVVHWHLDEKGWRFLDMEKQ	87
P48239_Xi.1/1-356	25	IY---------GALG--------CPFTHRAILARSLK------KLEPVLGLVLSHWQLDSKGARFLPAPHR	72
Q04806_Xi.1/1-366	40	LY---------VALP--------CPWAQRTLITRALK------GLAPIIGCSVAHWHLDDKGWRFLEEGDG	87

```
1A0F|A_E2KU21_Main.1/1-201              37  ---------------------------------------------------LENGDDYFAVNP-----KGQ  51
A9CII7_Main.1/1-208                     39  ----------------------------------------------------MKSPAYRLINP-----MAK  52
A9CJU4_Main.1/1-219                     43  ----------------------------------------------TDAPLNTLSPAFLAVNP-----MGT  62
A9CJU5_Main.1/1-206                     38  ----------------------------------------------------QRSEAFLKINP-----KGR  51
A9CLG1_Main.1/1-203                     40  ---------------------------------------------------GSGRKDPKNLHP-----EGK  54
Q7D3T0_Main.1/1-211                     37  ---------------------------------------------------TEGGQDYYDINP-----NGY  51
3C8E|A_YghU_D6JFD2_Main.2/1-284         83  ----------------------------------------------------QFSSGFVEVNP-----NSK  96
3GX0|A_YfcG_E7ICS5_Main.2/1-204         38  ----------------------------------------------------QFRPEFLRISP-----NNK  51
1G6W|A_E7KHF2_Main.2/1-234              52  ----------------------------------------------------HRAPEFVSVNP-----NAR  65
4ECI|A_Q02KA8_Main.2/1-204              39  ----------------------------------------------------QKAPEFLRINP-----NGR  52
4IKH|A_Q4KED9_Main.2/1-227              53  ----------------------------------------------------QMTPEFLSVSP-----NNK  66
Q03L82_Main.2/1-262                     85  ----------------------------------------------------QFGSDFVAINP-----NSK  98
Q8DTN7_Main.2/1-263                     85  ----------------------------------------------------QFGSDFVAINP-----NSK  98
A6B5E9_Main.2/1-285                     86  ----------------------------------------------------QFGSGFVAVNP-----NSK  99
Q2RXK8_Main.2/1-229                     38  ----------------------------------------------------QFAPEFLAISP-----NNR  51
Q4KH85_Main.2/1-213                     38  ----------------------------------------------------QFKPEFLKIAP-----NNR  51
1EEM|A_P78417_Main.3/1-237              54  ----------------------------------------------------NKPEWFFKKNP-----FGL  67
P34345_Main.3/1-250                     59  ----------------------------------------------------DKLEWYWTKHY-----QGK  72
Q2KDI2_Main.3/1-227                     38  ----------------------------------------------------NKPDWFLEISP-----LGK  51
Q8XW81_Main.3/1-224                     40  ----------------------------------------------------AKPDWFLALSP-----TGK  53
Q9H4Y5_Main.3/1-243                     58  ----------------------------------------------------NKPEWYYTKHP-----FGH  71
Q9VSL2_Main.3/1-241                     56  ----------------------------------------------------EKPEWLVEVSP-----LLK  69
Q9VSL3_Main.3/1-243                     56  ----------------------------------------------------DKPEWLLEKNP-----QGK  69
Q9VSL4_Main.3/1-250                     57  ----------------------------------------------------EKPEWYIDYSP-----LGK  70
Q9VSL5_Main.3/1-251                     57  ----------------------------------------------------EKPEWYKDFSP-----LGK  70
Q9VSL6_Main.3/1-254                     56  ----------------------------------------------------DKPEWFSLVSS-----STK  69
4HI7|A_B4KM86_Main.4/1-220              39  ----------------------------------------------------QHSEEYLKKNP-----QHT  52
3R2Q|A_B3HZI4_Main.9_NO_DSBR/1-202      35  ----------------------------------------------------NADNGVAQFNP-----LGK  48
A9CJG4_Main.9/1-198                     35  ----------------------------------------------------AEPATLMDNNP-----LGK  48
2R4V|A_O15247_Main.10_NO_DSBR/1-226     44  -----------------------------------------------------------DMTP-----GTN  50
Q8LE52_Main.10/1-258                    92  ----------------------------------------------------NKPEWFLKISP-----EGK  105
Q9FRL8_Main.10/1-213                    46  ----------------------------------------------------DKPQWFLDISP-----EGK  59
Q9FWR4_Main.10/1-213                    46  ----------------------------------------------------DKPQWFLDISP-----QGK  59
Q88LB2_Main.14/1-199                    37  ----------------------------------------------------NKPAELLALSP-----KGT  50
Q129E0_Main.14/1-230                    48  ----------------------------------------------------SKPPEMLAASS-----KGT  61
A9CKF2_Main.15/1-230                    36  ----------------------------------------------------ERRREFLALNP-----AGT  49
2YCD|A_A9CFJ9_Main.25/1-213             46  ----------------------------------------------------MKEASHLAYQP-----FGQ  59
B8H2E2_Main.25/1-217                    43  ----------------------------------------------------QDKTAYRAKQP-----FGQ  56
B7UV05_Main/1-220                       37  ----------------------------------------------------GQPAWYREISP-----LGR  50
1G7O|A_B3BP81_R4.1/1-215                35  -----------------------------------------------------DAETPTRMVG-----QKQ  47
D0W558_R4.1/1-213                       35  -----------------------------------------------------DEATPIGMIG-----AKQ  47
3PPU|A_YqjG_B3VQJ7_Xi.1/1-314           91  P----------------------------------------AADSDPLNNAQHVKDLYLKVKPDY-DGRFT  120
3R3E|A_C4ZR18_Xi.1/1-308                86  ------------------------------------------ATGDTLYQNEFLYQLYLHADPHY-SGRVT  113
C7GXD4_Xi.1/1-370                       88  LEDSEDFLEHWHDVAGGIRTAKEDSSKSFAEIKNDSQRFMVDATNEPHYGYKRISDLYYKSDPQY-SARFT  157
P48239_Xi.1/1-356                       73  PEKYKERFFTATGGIASAKL---DESEELGDVNNDSARLFVDGAFDPVENISRLSELYYLNDPKYPGTKFT  140
Q04806_Xi.1/1-366                       88  KTNERHWFDIAGGISSVNL----NTSTPVANIPNNAHRLLVDGTDEPHYGYKRLSDFYFKTKPDY-KGRFT  153
```

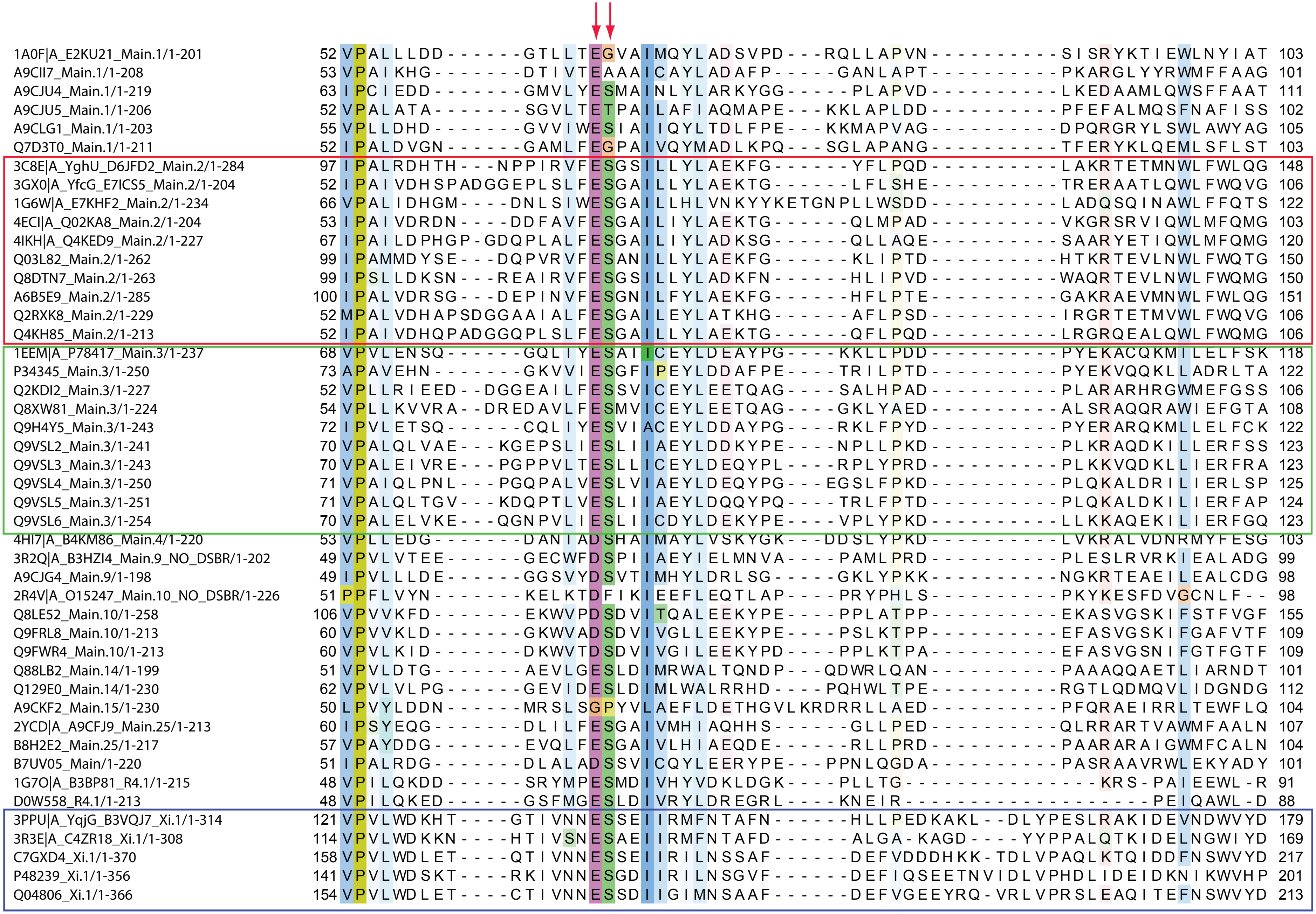
1A0F|A_E2KU21_Main.1/1-201 52 VPALLLDD------GTLLTEGVAIMQYLADSVPD---RQLLAPVN----------SISRYKTIEWLNYIAT 103
A9CII7_Main.1/1-208 53 VPAIKHG-------DTIVTEAAAICAYLADAFP----GANLAPT-----------PKARGLYYRWMFFAAG 101
A9CJU4_Main.1/1-219 63 IPCIEDD-------GMVLYESMAINLYLARKYGG-----PLAPVD----------LKEDAAMLQWSFFAAT 111
A9CJU5_Main.1/1-206 52 VPALATA-------SGVLTETPAILAFIAQMAPE---KKLAPLDD----------PFEFALMQSFNAFISS 102
A9CLG1_Main.1/1-203 55 VPLLDHD-------GVVIWESIAIIQYLTDLFPE---KKMAPVAG----------DPQRGRYLSWLAWYAG 105
Q7D3T0_Main.1/1-211 52 IPALDVGN------GAMLFEGPAIVQYMADLKPQ---SGLAPANG----------TFERYKLQEMLSFLST 103
3C8E|A_YghU_D6JFD2_Main.2/1-284 97 IPALRDHTH---NPPIRVFESGSILLYLAEKFG------YFLPQD----------LAKRTETMNWLFWLQG 148
3GX0|A_YfcG_E7ICS5_Main.2/1-204 52 IPAIVDHSPADGGEPLSLFESGAILLYLAEKTG------LFLSHE----------TRERAATLQWLFWQVG 106
1G6W|A_E7KHF2_Main.2/1-234 66 VPALIDHGM----DNLSIWESGAILLHLVNKYYKETGNPLLWSDD----------LADQSQINAWLFFQTS 122
4ECI|A_Q02KA8_Main.2/1-204 53 IPAIVDRDN----DDFAVFESGAILIYLAEKTG------QLMPAD----------VKGRSRVIQWLMFQMG 103
4IKH|A_Q4KED9_Main.2/1-227 67 IPAILDPHGP-GDQPLALFESGAILIYLADKSG------QLLAQE----------SAARYETIQWLMFQMG 120
Q03L82_Main.2/1-262 99 IPAMMDYSE---DQPVRVFESANILLYLAEKFG------KLIPTD----------HTKRTEVLNWLFWQTG 150
Q8DTN7_Main.2/1-263 99 IPSLLDKSN---REAIRVFESGSILLYLADKFN------HLIPVD----------WAQRTEVLNWLFWQMG 150
A6B5E9_Main.2/1-285 100 IPALVDRSG---DEPINVFESGNILFYLAEKFG------HFLPTE----------GAKRAEVMNWLFWLQG 151
Q2RXK8_Main.2/1-229 52 MPALVDHAPSDGGAAIALFESGAILEYLATKHG------AFLPSD----------IRGRTETMVWLFWQVG 106
Q4KH85_Main.2/1-213 52 IPAIVDHQPADGGQPLSLFESGAILLYLAEKTG------QFLPQD----------LRGRQEALQWLFWQMG 106
1EEM|A_P78417_Main.3/1-237 68 VPVLENSQ------GQLIYESAITCEYLDEAYPG----KKLLPDD----------PYEKACQKMILELFSK 118
P34345_Main.3/1-250 73 APAVEHN-------GKVVIESGFIPEYLDDAFPE----TRILPTD----------PYEKVQQKLLADRLTA 122
Q2KDI2_Main.3/1-227 52 VPLLRIEED--DGGEAILFESSVICEYLEETQAG----SALHPAD----------PLARARHRGWMEFGSS 106
Q8XW81_Main.3/1-224 54 VPLLKVVRA--DREDAVLFESMVICEYLEETQAG----GKLYAED----------ALSRAQQRAWIEFGTA 108
Q9H4Y5_Main.3/1-243 72 IPVLETSQ------CQLIYESVIACEYLDDAYPG----RKLFPYD----------PYERARQKMLLELFCK 122
Q9VSL2_Main.3/1-241 70 VPALQLVAE---KGEPSLIESLIIAEYLDDKYPE----NPLLPKD----------PLKRAQDKILLERFSS 123
Q9VSL3_Main.3/1-243 70 VPALEIVRE---PGPPVLTESLLICEYLDEQYPL----RPLYPRD----------PLKKVQDKLLIERFRA 123
Q9VSL4_Main.3/1-250 71 VPAIQLPNL---PGQPALVESLVIAEYLDEQYPG---EGSLFPKD----------PLQKALDRILIERLSP 125
Q9VSL5_Main.3/1-251 71 VPALQLTGV---KDQPTLVESLIIAEYLDQQYPQ----TRLFPTD----------PLQKALDKILIERFAP 124
Q9VSL6_Main.3/1-254 70 VPALELVKE---QGNPVLIESLIICDYLDEKYPE----VPLYPKD----------LLKKAQEKILIERFGQ 123
4HI7|A_B4KM86_Main.4/1-220 53 VPLLEDG-------DANIADSHAIMAYLVSKYGK---DDSLYPKD----------LVKRALVDNRMYFESG 103
3R2Q|A_B3HZI4_Main.9_NO_DSBR/1-202 49 VPVLVTEE------GECWFDSPIIAEYIELMNVA----PAMLPRD----------PLESLRVRKIEALADG 99
A9CJG4_Main.9/1-198 49 IPVLLLDE------GGSVYDSVTIMHYLDRLSG-----GKLYPKK----------NGKRTEAEILEALCDG 98
2R4V|A_O15247_Main.10_NO_DSBR/1-226 51 PPFLVYN-------KELKTDFIKIEEFLEQTLAP----PRYPHLS----------PKYKESFDVGCNLF-- 98
Q8LE52_Main.10/1-258 106 VPVVKFD-------EKWVPDSDVITQALEEKYPE----PPLATPP----------EKASVGSKIFSTFVGF 155
Q9FRL8_Main.10/1-213 60 VPVVKLD-------GKWVADSDVIVGLLEEKYPE----PSLKTPP----------EFASVGSKIFGAFVTF 109
Q9FWR4_Main.10/1-213 60 VPVLKID-------DKWVTDSDVIVGILEEKYPD----PPLKTPA----------EFASVGSNIFGTFGTF 109
Q88LB2_Main.14/1-199 51 VPVLDTG-------AEVLGESLDIMRWALTQNDP---QDWRLQAN----------PAAAQQAETLIARNDT 101
Q129E0_Main.14/1-230 62 VPVLVLPG------GEVIDESLDIMLWALRRHD----PQHWLTPE----------RGTLQDMQVLIDGNDG 112
A9CKF2_Main.15/1-230 50 LPVYLDDN------MRSLSGPYVLAEFLDETHGVLKRDRRLLAED----------PFQRAEIRRLTEWFLQ 104
2YCD|A_A9CFJ9_Main.25/1-213 60 IPSYEQG-------DLILFESGAIVMHIAQHHS------GLLPED----------QLRRARTVAWMFAALN 107
B8H2E2_Main.25/1-217 57 VPAYDDG-------EVQLFESGAIVLHIAEQDE------RLLPRD----------PAARARAIGWMFCALN 104
B7UV05_Main/1-220 51 IPALRDG-------DLALADSSVICQYLEERYPE---PPNLQGDA----------PASRAAVRWLEKYADY 101
1G7O|A_B3BP81_R4.1/1-215 48 VPILQKDD------SRYMPESMDIVHYVDKLDGK----PLLTG---------------KRS-PAIEEWL-R 91
D0W558_R4.1/1-213 48 VPILQKED------GSFMGESLDIVRYLDREGRL----KNEIR-------------------PEIQAWL-D 88
3PPU|A_YqjG_B3VQJ7_Xi.1/1-314 121 VPVLWDKHT----GTIVNNESSEIIRMFNTAFN------HLLPEDKAKL--DLYPESLRAKIDEVNDWVYD 179
3R3E|A_C4ZR18_Xi.1/1-308 114 VPVLWDKKN----HTIVSNESAEIIRMFNTAFD------ALGA-KAGD----YYPPALQTKIDELNGWIYD 169
C7GXD4_Xi.1/1-370 158 VPVLWDLET----QTIVNNESSEIIRILNSSAF------DEFVDDDHKK-TDLVPAQLKTQIDDFNSWVYD 217
P48239_Xi.1/1-356 141 VPVLWDSKT----RKIVNNESGDIIRILNSGVF------DEFIQSEETNVIDLVPHDLIDEIDKNIKWVHP 201
Q04806_Xi.1/1-366 154 VPVLWDLET----CTIVNNESSDIIGIMNSAAF------DEFVGEEYRQ-VRLVPRSLEAQITEFNSWVYD 213

```
1A0F|A_E2KU21_Main.1/1-201                104 ELHKGF-TPLFRPD----------------------TP-----------------EEYKPTVRAQLEK 131
A9CII7_Main.1/1-208                       102 PLEMAA-SMKAMGF--------------------EVPK-----------------EKLRMAGCGSYAD 131
A9CJU4_Main.1/1-219                       112 EIETNS-LKISSAI----------------AEGLAESDA---------------GKAVIDVAARLLKR 147
A9CJU5_Main.1/1-206                       103 TVHVAH-AHGRRGS----------------RWANEDSSL----------------ADMKARVPKNMGE 137
A9CLG1_Main.1/1-203                       106 VVEPVL-IMQAAGI----------------------------------------SHPFVDFTFRSPAE 132
Q7D3T0_Main.1/1-211                       104 EIHKGF-IPLLYAK----------------------QA-----------------GAYLETATPKLEK 131
3C8E|A_YghU_D6JFD2_Main.2/1-284           149 A-APFLGGGFGHFY----------------HYAP-VKI-----------------EYAINRFTMEAKR 181
3GX0|A_YfcG_E7ICS5_Main.2/1-204           107 GLGPML-GQNHHFN----------------HAAP-QTI-----------------PYAIERYQVETQR 139
1G6W|A_E7KHF2_Main.2/1-234                123 GHAPMI-GQALHFR----------------YFHS-QKI-----------------ASAVERYTDEVRR 155
4ECI|A_Q02KA8_Main.2/1-204                104 GVGPMQ-GQANVFF----------------RYFP-EKL-----------------QGAIDRYQHETRR 136
4IKH|A_Q4KED9_Main.2/1-227                121 GIGPMF-GQVGFFN----------------KFAGREYED----------------KRPLERYVNEAKR 155
Q03L82_Main.2/1-262                       151 A-APFLGGGFGHFF----------------HYAP-EKI-----------------EYAVNRFAMEAKR 183
Q8DTN7_Main.2/1-263                       151 A-APFVGGGFGHFF----------------SYAP-EKL-----------------EYPINRFTMETKR 183
A6B5E9_Main.2/1-285                       152 S-APYLGGGFGHFY----------------AYAP-EKF-----------------EYPINRFTMEVKR 184
Q2RXK8_Main.2/1-229                       107 GLGPMA-GQAHHFR----------------QYAP-EPV-----------------PYAIERYTNEVNR 139
Q4KH85_Main.2/1-213                       107 GLGPMA-GQNHHFS----------------QFAP-EKI-----------------PYAIKRYVDETAR 139
1EEM|A_P78417_Main.3/1-237                119 VPSLVG-SFIRSQN-----------------------------------------KEDYAGLKEEFRK 144
P34345_Main.3/1-250                       123 VAHAVP-LLFAVMR----------------------------------------DRTLKDEKQRKVFE 149
Q2KDI2_Main.3/1-227                       107 VLSDLW-GYETATE-----------------------------------------VEQLEAKRKALIA 132
Q8XW81_Main.3/1-224                       109 TLADAW-QFLNASD-----------------------------------------RTTSDDKRAAFRK 134
Q9H4Y5_Main.3/1-243                       123 VPHLTK-ECLVALR----------------------------------------CGRECTNLKAALRQ 149
Q9VSL2_Main.3/1-241                       124 ITSAFI-NIL-----------------------------------------------VQGTGLEDYWT 143
Q9VSL3_Main.3/1-243                       124 VLGAFF-------------------------------------------------KASDGGDLEPFWS 142
Q9VSL4_Main.3/1-250                       126 AVSAIY-PVLFT-------------------------------------------KNPPADAIKNFET 149
Q9VSL5_Main.3/1-251                       125 VVSAIY-PVLTCN------------------------------------------PNAPKDAIPNFEN 149
Q9VSL6_Main.3/1-254                       124 FINAFY-YLLLH-------------------------------------------DNPEQLVDTDHYA 147
4HI7|A_B4KM86_Main.4/1-220                104 VVFANALRSLAKMI----------------LFLG-KTE-------------------VPQERIDAITE 135
3R2Q|A_B3HZI4_Main.9_NO_DSBR/1-202        100 IMDAGL-VSVREQA----------------RPAA-QQS-----------------EDELLRQREKINR 132
A9CJG4_Main.9/1-198                        99 IMDCLL-AIVYERR----------------FRPEDKIH-----------------QPWIDKQWSKVVR 132
2R4V|A_O15247_Main.10_NO_DSBR/1-226        99 ---AKF-SAYIK-----------------------NTQ-----------------KEANKNFEKSLLK 122
Q8LE52_Main.10/1-258                      156 LKS----------------------------------------------------KDSGDGTEQVLLD 171
Q9FRL8_Main.10/1-213                      110 LKS----------------------------------------------------KDANDGSEKALVD 125
Q9FWR4_Main.10/1-213                      110 LKS----------------------------------------------------KDSNDGSEHALLV 125
Q88LB2_Main.14/1-199                      102 TFKAHV-NLYKYAE---------------------RYP-----------------EHSREHYRQQAEA 130
Q129E0_Main.14/1-230                      113 AFKRNL-DRYKYPN----------------RYLEEAAEETA-----------GDEAVFAEAHRTAGAA 152
A9CKF2_Main.15/1-230                      105 KMENDV-TRPLVRE----------------RVYKLQMTAAQGG--------GPPDSKLLRTARNNIRQ 147
2YCD|A_A9CFJ9_Main.25/1-213               108 TIEPSI-LNFTTVW----------------LFERNEPWH----------------EARLARTKEQLLK 142
B8H2E2_Main.25/1-217                      105 TLEVPL-LQLAELN----------------IFSAGQAWT----------------EGARPKVERWVRQ 139
B7UV05_Main/1-220                         102 EIAPLA-TLTIFRN----------------RILKPAMGQAC------------EENDVRRALEEKLPA 140
1G7O|A_B3BP81_R4.1/1-215                   92 KVNGYA-NKLLLPRFAKSAFDEFSTPAARKYFVDKKEASAGNF---------ADLLAHSDGLIKNISD 149
D0W558_R4.1/1-213                          89 KVGSYN-NKLVQPRTVKIGLPEFATAEAVKYFTDKKEKNIGSF---------SANLNKTAQYLDRIHE 146
3PPU|A_YqjG_B3VQJ7_Xi.1/1-314             180 TVNNGV-YKSGF---------------------AST-------------------QKAYEAAVIPLFE 206
3R3E|A_C4ZR18_Xi.1/1-308                  170 TVNNGV-YKAGF---------------------ATS-------------------QEAYDEAVAKVFE 196
C7GXD4_Xi.1/1-370                         218 SINNGV-YKTGF---------------------AEKAEVYESEVNNVFEHLDKVEKILSDKYSKLKAK 263
P48239_Xi.1/1-356                         202 KINLGV-YKVGL---------------------AENGKIYETEVK-TLFENLQKMECVLKENYKRLEE 246
Q04806_Xi.1/1-366                         214 KINNGV-YKAGF---------------------AECAEVYEREVTSLFQYLDKLENLLDKKYTDLEAE 259
```

1A0F|A_E2KU21_Main.1/1-201	132	KLQYVNEALKDE------------------	HWICGQRFTIAD	AYLFTVLRWAY--AVK-------------	169
A9CII7_Main.1/1-208	132	VMNTLERAVSEN------------------	RFIAGDLFTAAD	VYVGAHVGWGL--HF--------------	168
A9CJU4_Main.1/1-219	148	PLRVLEQHLATH------------------	DYLVGDRFTVAD	LNVAEIVRYAQ--GHQ-------------	185
A9CJU5_Main.1/1-206	138	CFELIEHGMLHG------------------	PWVLGTAYSVAD	PYLFVMSGWLE--SDG-------------	175
A9CLG1_Main.1/1-203	133	LAARLETALKDC------------------	PYLMGERYTAVD	LLLHSPFAWCP------------------	167
Q7D3T0_Main.1/1-211	132	RYAWIDNLLASQ------------------	RFLMGENFTVAD	AYLFALTSWGQ--ASWLKSYYG-----AD	177
3C8E|A_YghU_D6JFD2_Main.2/1-284	182	LLDVLDKQLAQH------------------	KFVAGDEYTIAD	MAIWPWFGNVV-LGGVYDAAE-------F	226
3GX0|A_YfcG_E7ICS5_Main.2/1-204	140	LYHVLNKRLENS------------------	PWLGGENYSIAD	IACWPWVNAWT--RQ-------------R	177
1G6W|A_E7KHF2_Main.2/1-234	156	VYGVVEMALAE------RREALVMELDDYP	VWLVGDKLTIAD	LAFVPWNNVVD--RI-------------G	205
4ECI|A_Q02KA8_Main.2/1-204	137	LYEVLDGRLGEA------------------	EYLAG-DYSIAD	IATYPWVRIHD--WS-------------G	173
4IKH|A_Q4KED9_Main.2/1-227	156	LLGVLDKHLGGR------------------	EWIMGERYTIAD	IATFPWIRNLI--GFYEAGE--------L	198
Q03L82_Main.2/1-262	184	QLDLLDKELATK------------------	PYISGDEYTIAD	IAIWSWYGRLAQDKIWDKAGI-------F	229
Q8DTN7_Main.2/1-263	184	QLDLLNKELANK------------------	PYIAGEDYTIAD	IAIWSWYGRLAQDALYEGAYK-------F	229
A6B5E9_Main.2/1-285	185	QLDVLDKRLANN------------------	AFLGGDEYSIAD	IATWPWYGNLV-LGRAYDAAE-------F	229
Q2RXK8_Main.2/1-229	140	LYGVMNKRLADR------------------	DYLAG-AYSIAD	MASWPWVVSHA--NQ-------------G	176
Q4KH85_Main.2/1-213	140	LYGVLDRRLADR------------------	KFVAGSDYSIAD	MAIYPWIASYK--KQ-------------S	177
1EEM|A_P78417_Main.3/1-237	145	EFTKLEEVLTN------KKT----------	TFFGGNSISMID	YLIWPWFERLE--AMKL-----------N	186
P34345_Main.3/1-250	150	VLKQAENLLAN-------------------	DFYAGSQPGYPD	YLSFPFFEKIW--WSASLDGVVDLPTIEF	199
Q2KDI2_Main.3/1-227	133	KFATLEGVLADG------------------	PYFSGSSFSLVD	AVFAPIFRYFD--VFERLGD--------S	175
Q8XW81_Main.3/1-224	135	KLQQLEKAVAAE------------------	PYFSGSTFSMVD	AVYAPLFRYFD--ILDPKVS--------Q	177
Q9H4Y5_Main.3/1-243	150	EFSNLEEILEY------QNT----------	TFFGGTCISMID	YLLWPWFERLD--VYGI-----------L	191
Q9VSL2_Main.3/1-241	144	ALDIFEEELTK------RGT----------	PYFGGNKPGFVD	YMIWPWFERLS--VIELKLQKE-----YN	191
Q9VSL3_Main.3/1-243	143	GLDIYERELAR------RGT----------	EFFGGEQTGILD	YMIWPWCERLE--LLKLQRGED-----YN	190
Q9VSL4_Main.3/1-250	150	ALDVFEQEITK------RGT----------	PYFGGNKIGIAD	YMIWPWFERFP--ALKYTLDEP-----YE	197
Q9VSL5_Main.3/1-251	150	ALDVFEVELGK------RGT----------	PYFAGQHIGIVD	YMIWPWFERFP--SMKINTEQK-----YE	197
Q9VSL6_Main.3/1-254	148	GLVVYEEELKR------RCT----------	KFFGGDSPGMLD	YMMWPWCERFD--SLKYTFEQK-----FE	195
4HI7|A_B4KM86_Main.4/1-220	136	AYDFVEAFFKDQ------------------	TYVAGNQLTIAD	FSLISSISSLV--AF-------------V	173
3R2Q|A_B3HZI4_Main.9_NO_DSBR/1-202	133	SLDVLEGYLVD-------------------	GTLKTDTVNLAT	IAIACAVGYLN--FRRVA----------P	172
A9CJG4_Main.9/1-198	133	GLDHLNANLP--------------------	--KTGKKLHGGH	FALAAMIGYLD--LRFAG-----------	168
2R4V|A_O15247_Main.10_NO_DSBR/1-226	123	EFKRLDDYLNTPLLDEIDPDSAEEPPVSRR	LFLDGDQLTLAD	CSLLPKLNIIK--VAAKKYRD-------F	184
Q8LE52_Main.10/1-258	172	ELTTFNDYIKDN-----------------G	PFINGEKISAAD	LSLAPKLYHMK--IALGHYKN-------W	216
Q9FRL8_Main.10/1-213	126	ELEALENHLKT------HSG----------	PFVAGEKITAVD	LSLAPKLYHLE--VALGHYKN-------W	171
Q9FWR4_Main.10/1-213	126	ELEALENHLKS------HDG----------	PFIAGERVSAVD	LSLAPKLYHLQ--VALGHFKS-------W	171
Q88LB2_Main.14/1-199	131	WLAELEGLLAGR------------------	AYLLADHPSMAD	AALLPLMRQFA--GVEPQW----------	171
Q129E0_Main.14/1-230	153	WLGRLELMLEQH------------------	GCLFGAQASLAD	MALLPFVRQFA--HTDAAW----------	193
A9CKF2_Main.15/1-230	148	HIKYLEWLAGSR------------------	TWLAGDRLSYAD	LAAAAGVSVLD--YLGE------------	186
2YCD|A_A9CFJ9_Main.25/1-213	143	RLDELSAWLGDR------------------	EWLEG-SFSAAD	ILMICVLRRLE--SS--------------	178
B8H2E2_Main.25/1-217	140	RLGQLAARLGDK------------------	TYLDGETFTAGD	LLVADVLRQAP--DAML------------	178
B7UV05_Main/1-220	141	HFDYLENHLDGR------------------	AFFVGERLTLAD	LAIASQLVNLR--HAGE-----------N	180
1G7O|A_B3BP81_R4.1/1-215	150	DLRALDKLIVK-------------------	PNAVNGELSEDD	IQLFPLLRNLT--LVA------------G	187
D0W558_R4.1/1-213	147	DLNDLDGLMHEV------------------	SDGINGEIGMED	ILIFPILRNLT--VVR------------G	185
3PPU|A_YqjG_B3VQJ7_Xi.1/1-314	207	SLDRLEKMLEGQ------------------	DYLIGGQLTEAD	IRLFVTIVRFD--PVYVTHFKC-----NL	252
3R3E|A_C4ZR18_Xi.1/1-308	197	SLARLEQILGQH------------------	RYLTGNQLTEAD	IRLWTTLVRFD--PVYVTHFKC-----DK	242
C7GXD4_Xi.1/1-370	264	YGEEDRQKILGE------------------	FFTVGDQLTEAD	IRLYTTVIRFD--PVYVQHFKC-----NF	309
P48239_Xi.1/1-356	247	QFSGNKQKILAK------------------	YFVLGQRLTEAD	IRLYPSIIRFD--VVYVQHFKC-----NL	292
Q04806_Xi.1/1-366	260	YGKNNKDKILDR------------------	YFAIGDTLTEAD	VRLYPTIVRFD--VVYHQHFKC-----NL	305

```
1A0F|A_E2KU21_Main.1/1-201          170 LNL-EGLEHIAAFMQRMA-ERPEVQDALSAEGL--K----------------------------------- 201
A9CII7_Main.1/1-208                 169 GTI-EKRPAFTDYMAHLT-DRPAFKRAAQLDEE--AAK----------------------ELQAAG----- 208
A9CJU4_Main.1/1-219                 186 PLF-DAHPALKAWLARCQ-ARTAFKSMWDSRTA--EAA--------------------------------- 219
A9CJU5_Main.1/1-206                 176 VDI-ARFPKVHDHFRRMN-DRPAVQRALEGERG-------------------------------------- 206
A9CLG1_Main.1/1-203                 168 AAT-PESDVVKAWIERCG-ERPSLQWTANYDAC--TVVAA------------------------------- 203
Q7D3T0_Main.1/1-211                 178 IHF-DDLLNLRGWYERVK-ERDAVKQSILEEGL--SLS--------------------------------- 211
3C8E|A_YghU_D6JFD2_Main.2/1-284     227 LDA-GSYKHVQRWAKEVG-ERPAVKRGRIVNRT--NGP-LNEQLHERHDASDFETNTEDKRQG-------- 284
3GX0|A_YfcG_E7ICS5_Main.2/1-204     178 IDL-AMYPAVKNWHERIR-SRPATGQALL------------------------------------------ 204
1G6W|A_E7KHF2_Main.2/1-234          206 INIKIEFPEVYKWTKHMM-RRPAVIKALRG----------------------------------------- 234
4ECI|A_Q02KA8_Main.2/1-204          174 VAV-DGLDNLQRWIAAIE-ARPAVQRGLLVPRR-------------------------------------- 204
4IKH|A_Q4KED9_Main.2/1-227          199 VGI-DNFPEVKRVLAKFV-ARPAVIRGLEIP---------------------------------------- 227
Q03L82_Main.2/1-262                 230 LDV-KEYKHLQAWTEKIA-NRPAVKRGLEVEYK--EI---------------------------------- 262
Q8DTN7_Main.2/1-263                 230 LAL-GTYQHLLDWTERIA-QRPAVKRALEVDYK--AIK--------------------------------- 263
A6B5E9_Main.2/1-285                 230 LDV-ESYKNVVRWAKAID-EREGVKRGRIVNRS--WGE-EWEQLAERHSAVDIDNVL--KLKP-------- 285
Q2RXK8_Main.2/1-229                 177 QSL-DDFPNLKRWFDAIG-ARPAVQRGMALGAD--LRE-ANANTTPEARAVLFGQKAR------------- 229
Q4KH85_Main.2/1-213                 178 QKL-EDFPNLQRWFNSIQ-ARPATERAYALVEQ--VNPAS------------------------------- 213
1EEM|A_P78417_Main.3/1-237          187 ECV-DHTPKLKLWMAAMK-EDPTVSALLTSEKD--WQGFLELYLQN----SPEACDYGL------------ 237
P34345_Main.3/1-250                 200 PGE-EEYPKLTKWFQKMI-SSDVVQSVTQSLEH--GAAFMNAYATH----QELNYDLGL------------ 250
Q2KDI2_Main.3/1-227                 176 GIF-AGLERVTRWRIALG-ERRSVKDAVGEDYPQRLMEFLDKHESI----LLRLPAAA------------- 227
Q8XW81_Main.3/1-224                 178 PIF-EGLPRVKAWRAALG-ARESVIAAVGEDYAERFGQHLRLHQAL----LAN------------------ 224
Q9H4Y5_Main.3/1-243                 192 DCV-SHTPALRLWISAMK-WDPTVCALLMDKSI--FQGFLNLYFQN---NPNAFDF--GLC---------- 243
Q9VSL2_Main.3/1-241                 192 FNE-SRFPKITKWIALLK-ADSVVQSFYATPEQ--HNEFWRTRKAG---NAN-YDLLA------------- 241
Q9VSL3_Main.3/1-243                 191 YDQ-SRFPQLTLWLERMK-RDPAVMAFYMEAEV--QAEFLRTRSLG---RPN-YNL--LVKDA-------- 243
Q9VSL4_Main.3/1-250                 198 LDK-TRYQNLLKWRDLVA-QDEAVKATALDARI--HAKFMKTRHEN----KPD-YDV--AFQPL------- 250
Q9VSL5_Main.3/1-251                 198 LDT-KRFEKLLKWRDLMT-QDEVVQKTALDVQL--HAEFQKSKTLG---NPQ-YDI--AFKGTP------ 251
Q9VSL6_Main.3/1-254                 196 LSP-ERFPTLIKWRDLMI-QDRAVKCFYLDGQT--HAKYMNSRRSG----QAD-YNM--LYNEAKRVKLG- 254
4HI7|A_B4KM86_Main.4/1-220          174 PVDAAKYPKLSAWIKRLE-QLPYYAENSTGAQQ--FVAAVKSKPFT----VVGA----------------- 220
3R2Q|A_B3HZI4_Main.9_NO_DSBR/1-202  173 GWX-VDRPHLVKLVENLF-SRESFARTEPPKA---------------------------------------- 202
A9CJG4_Main.9/1-198                 169 -EWAEGRDALAAWPETFG-KRFDAYAEMKAAA---------------------------------------- 198
2R4V|A_O15247_Main.10_NO_DSBR/1-226 185 DIP-AEFSGVWRYLHNAY-AREEFTHTCPEDKE--IENTYANVAKQ------------------------- 226
Q8LE52_Main.10/1-258                217 SVP-DSLPFVKSYMENVF-SRESFTNTRAETED--VIAGWRPKVMG------------------------- 258
Q9FRL8_Main.10/1-213                172 SVP-ESLTSVRNYAKALF-SRESFENTKAKKEI--VVAGWESKVNA------------------------- 213
Q9FWR4_Main.10/1-213                172 SVP-ESFPHVHNYMKTLF-SLDSFEKTKTEEKY--VISGWAPKVNP------------------------- 213
Q88LB2_Main.14/1-199                172 -FAEAAYPRVRSWLEGWL-ASELFKAIMVR------------------------------------------ 199
Q129E0_Main.14/1-230                194 -FTAQPWPRLQAWLAGFE-ASALYQSVMEKHAP--WRA-ASE----------------------------- 230
A9CKF2_Main.15/1-230                187 INW-LEAPIAKEWYQRVK-SRPSFRPFLSERIP--RLAPSSHYADLDF----------------------- 230
2YCD|A_A9CFJ9_Main.25/1-213         179 GIL-KDYGNLLAYVERGK-ARPAFKRAFDAQLA--VFTA-------------------------------- 213
B8H2E2_Main.25/1-217                179 ----AETPTLVAYRDRCT-ARPAFQKALAAQLA--DLN----------------------GPPPPGWG--- 217
B7UV05_Main/1-220                   181 LDE-QRWPALAAHFARML-ERPAMQALLPGERR--TLDKLAAKA--------------------------- 220
1G7O|A_B3BP81_R4.1/1-215            188 INW---PSRVADYRDNMA--KQTQINL--------LSSMAI------------------------------ 215
D0W558_R4.1/1-213                   186 IEW---PQKVMGYLMTMS--EKSGVPL--------YFDRAL------------------------------ 213
3PPU|A_YqjG_B3VQJ7_Xi.1/1-314       253 RTIRDGYPNLHRWMRKLYWGNPAFKDTCNFEHI--KTH-YFWSHTF---INPHRIVP--IGPIPDILPLD- 314
3R3E|A_C4ZR18_Xi.1/1-308            243 HRIS-DYLNLYGFLRDIY-QMPGIAETVNFDHI--RNH-YFRSHKT---INPTGIIS--IGPWQDLDEPHG 303
C7GXD4_Xi.1/1-370                   310 TSIRAGYPFIHLWVRNLYWNYDAFRYTTDFDHI--KLH-YTRSHTR---INPLGITP--LGPKPDIRPL-- 370
P48239_Xi.1/1-356                   293 KTIRDGFPYLHLWLINLYWNYAEFRFTTDFNHI--KLF-YIRMEVSRNKINQFGIVP--LGPKPDISRL-- 356
Q04806_Xi.1/1-366                   306 ATIRDDYSRIHTWLKNIYWRHEAFQRTTDFTHI--KLG-YTRSQPR---VNPIGITP--LGPKPDIRPP-- 366
```

1A0F|A_E2KU21_Main.1/1-201	- - - - -
A9CII7_Main.1/1-208	- - - - -
A9CJU4_Main.1/1-219	- - - - -
A9CJU5_Main.1/1-206	- - - - -
A9CLG1_Main.1/1-203	- - - - -
Q7D3T0_Main.1/1-211	- - - - -
3C8E|A_YghU_D6JFD2_Main.2/1-284	- - - - -
3GX0|A_YfcG_E7ICS5_Main.2/1-204	- - - - -
1G6W|A_E7KHF2_Main.2/1-234	- - - - -
4ECI|A_Q02KA8_Main.2/1-204	- - - - -
4IKH|A_Q4KED9_Main.2/1-227	- - - - -
Q03L82_Main.2/1-262	- - - - -
Q8DTN7_Main.2/1-263	- - - - -
A6B5E9_Main.2/1-285	- - - - -
Q2RXK8_Main.2/1-229	- - - - -
Q4KH85_Main.2/1-213	- - - - -
1EEM|A_P78417_Main.3/1-237	- - - - -
P34345_Main.3/1-250	- - - - -
Q2KDI2_Main.3/1-227	- - - - -
Q8XW81_Main.3/1-224	- - - - -
Q9H4Y5_Main.3/1-243	- - - - -
Q9VSL2_Main.3/1-241	- - - - -
Q9VSL3_Main.3/1-243	- - - - -
Q9VSL4_Main.3/1-250	- - - - -
Q9VSL5_Main.3/1-251	- - - - -
Q9VSL6_Main.3/1-254	- - - - -
4HI7|A_B4KM86_Main.4/1-220	- - - - -
3R2Q|A_B3HZI4_Main.9_NO_DSBR/1-202	- - - - -
A9CJG4_Main.9/1-198	- - - - -
2R4V|A_O15247_Main.10_NO_DSBR/1-226	- - - - -
Q8LE52_Main.10/1-258	- - - - -
Q9FRL8_Main.10/1-213	- - - - -
Q9FWR4_Main.10/1-213	- - - - -
Q88LB2_Main.14/1-199	- - - - -
Q129E0_Main.14/1-230	- - - - -
A9CKF2_Main.15/1-230	- - - - -
2YCD|A_A9CFJ9_Main.25/1-213	- - - - -
B8H2E2_Main.25/1-217	- - - - -
B7UV05_Main/1-220	- - - - -
1G7O|A_B3BP81_R4.1/1-215	- - - - -
D0W558_R4.1/1-213	- - - - -
3PPU|A_YqjG_B3VQJ7_Xi.1/1-314	- - - - -
3R3E|A_C4ZR18_Xi.1/1-308	304 R D V R F 308
C7GXD4_Xi.1/1-370	- - - - -
P48239_Xi.1/1-356	- - - - -
Q04806_Xi.1/1-366	- - - - -
