# Supplementary material for: Large-Scale Determination of Sequence, Structure, and Function Relationships in Cytosolic Glutathione Transferases across the Biosphere
Source: PLoS Biol. 2014 Apr 22;12(4):e1001843. doi: 10.1371/journal.pbio.1001843 (PMC3995644; doi:10.1371/journal.pbio.1001843)
Supplement: Table S1 — High throughput assays used in this work. The assays for cytGST-like activity used in this work are shown, categorized by reaction type, and the substrates used are indicated. References for the methods are also given. (DOCX) [file pbio.1001843.s005.docx]

**Table S1. High throughput assays used in this work.**

| Activity/Reaction Type | Substrate | References |
| --- | --- | --- |
| 1. Nucleophilic Aromatic Substitution | CDNB | *(*[*1-3*](#_ENREF_1)*)* |
| 2. Nucleophilic Aromatic Substitution | 4-nitroquinoline 1-oxide | *(*[*4*](#_ENREF_4)*)* |
| 3. Nucleophilic Substitution | Iodomethane | *(*[*5*](#_ENREF_5)*)* |
| 4. Nucleophilic Substitution | Iodoethane | *(*[*5*](#_ENREF_5)*)* |
| 5. Nucleophilic Substitution | Iodoacetate | *(*[*5*](#_ENREF_5)*)* |
| 6. Nucleophilic Substitution | Iodoacetamide | *(*[*5*](#_ENREF_5)*)* |
| 7. Conjugate Addition | Ethacrynic acid | *(*[*1*](#_ENREF_1)*,* [*3*](#_ENREF_3)*)* |
| 8. Conjugate Addition | *trans*-2-nonenal | *(*[*1*](#_ENREF_1)*,* [*6*](#_ENREF_6)*)* |
| 9. Nucleophilic Addition | Cyclohexyl isothiocyanate | *(*[*7*](#_ENREF_7)*)* |
| 10. Nucleophilic Addition | Phenethyl isothiocyanate | *(*[*7*](#_ENREF_7)*)* |
| 11. Nucleophilic Addition | Propyl isothiocyanate | *(*[*7*](#_ENREF_7)*)* |
| 12. Thiolysis | 4-nitrophenyl acetate | *(*[*3*](#_ENREF_3)*)* |
| 13. Disulfide Bond Reductase | 2-hydroxyethyl disulfide | *(*[*8*](#_ENREF_8)*)* |
| 14. Disulfide Bond Reductase | Cysteine disulfide | *(*[*8*](#_ENREF_8)*)* |
| 15. Disulfide Bond Reductase | Furfuryl disulfide | *(*[*8*](#_ENREF_8)*)* |
| 16. Disulfide Bond Reductase | Pantethine disulfide | *(*[*8*](#_ENREF_8)*)* |
| 17. Peroxidase | Benzoyl peroxide | *(*[*9-11*](#_ENREF_9)*)* |
| 18. Peroxidase | Cumene hydroperoxide | *(*[*9-11*](#_ENREF_9)*)* |
| 19. Peroxidase | t-butyl hydroperoxide | *(*[*9-11*](#_ENREF_9)*)* |
| 20. Hydrolytic Dehalogenation | Diiodomethane | *(*[*5*](#_ENREF_5)*)* |

**References**

1. Mannervik, B., and Jemth, P. (2001) Measurement of glutathione transferases, *Current protocols in toxicology / editorial board, Mahin D. Maines (editor-in-chief) ... [et al.]* *Chapter 6*, Unit6 4.

2. Habig, W. H., Pabst, M. J., and Jakoby, W. B. (1974) Glutathione S-transferases. First enzymic step in mercapturic acid formation, *Journal of Biological Chemistry* *249*, 7130-7139.

3. Habig, W. H., and Jakoby, W. B. (1981) Assays for differentiation of glutathione S-transferases, *Methods Enzymol* *77*, 398-405.

4. Stanley, J. S., and Benson, A. M. (1988) The conjugation of 4-nitroquinoline 1-oxide, a potent carcinogen, by mammalian glutathione transferases. 4-Nitroquinoline 1-oxide conjugation by human, rat and mouse liver cytosols, extrahepatic organs of mice and purified mouse glutathione transferase isoenzymes, *The Biochemical journal* *256*, 303-306.

5. Kurtovic, S., Jansson, R., and Mannervik, B. (2007) Colorimetric endpoint assay for enzyme-catalyzed iodide ion release for high-throughput screening in microtiter plates, *Archives of biochemistry and biophysics* *464*, 284-287.

6. Aalin, P., Danielson, U. H., and Mannervik, B. (1985) 4-Hydroxyalk-2-enals are substrates for glutathione transferase, *FEBS Letters* *179*, 267-270.

7. Kolm, R. H., Danielson, U. H., Zhang, Y., Talalay, P., and Mannervik, B. (1995) Isothiocyanates as substrates for human glutathione transferases: structure-activity studies, *The Biochemical journal* *311 ( Pt 2)*, 453-459.

8. Holmgren, A., and Aaslund, F. (1995) Glutaredoxin, *Methods in Enzymology* *252*, 283-292.

9. Mannervik, B. (1985) Glutathione peroxidase, *Methods in Enzymology* *113*, 490-495.

10. Flohe, L., and Guenzler, W. A. (1984) Assays of glutathione peroxidase, *Methods in Enzymology* *105*, 114-121.

11. Edwards, R., and Dixon, D. P. (2005) Plant glutathione transferases, *Methods in Enzymology* *401*, 169-186.
