# Supplementary material for: Large-Scale Determination of Sequence, Structure, and Function Relationships in Cytosolic Glutathione Transferases across the Biosphere
Source: PLoS Biol. 2014 Apr 22;12(4):e1001843. doi: 10.1371/journal.pbio.1001843 (PMC3995644; doi:10.1371/journal.pbio.1001843)
Supplement: Text S1 — How thresholds for viewing networks were chosen. (PDF) [file pbio.1001843.s010.pdf]

Text File S1. How thresholds for viewing networks were chosen.

The Level 1 and Level 2 thresholds were chosen visually to roughly subdivide the superfamily into manageable groups. The Level 1 threshold we chose (*E*-value $1x10^{-13}$) was that at which the AMPS classes form a single cluster cleanly separated from the largest "Main" cluster. This is consistent with the conclusions of the primary literature describing proteins of these relatively better-studied classes. These groupings persist through several more stringent cutoffs and probably reflect early phylogenetic divergences in the superfamily.

In choosing the Level 2 threshold for subdividing the superfamily, more stringent thresholds were stepped through and visually examined. Good separation by canonical class occurred at an *E*-value cutoff of $1x10^{-25}$. It was observed that at thresholds more stringent than this threshold even more of the canonical classes became separated from other classes; however, it was also seen that some canonically classified nodes began to separate from others of their own class, resulting in network views that have lost some information about the patterns by which the most diverse nodes assigned to a particular canonical class relate to other nodes of the same class (data not shown). The Level 2 threshold we ultimately chose to show in the representative network Figures was thus a compromise between obtaining good separation between canonical classes and keeping previously classified sequences together; i.e., keeping historically assigned classes together was favored over separation. The resulting Level 2 subgroupings show how

annotated proteins relate to each other framed as much as much as possible in terms of the classes that had been defined over decades of cytGST research.

We also needed to choose an appropriate threshold for viewing the details of each Level 2 subgroup as a separate, full network (where, in contrast to representative networks, each node corresponds to a single sequence). Here, since the membership of each subgroup is defined by the $1x10^{-25}$ cutoff, each subgroup includes most of the sequences in one or more discrete canonical class, along with many sequences of unassigned class. For visualizing subclustering within the full networks, we chose a cutoff that showed these sequences separated as well as possible by canonical class. As illustrated in Figure 7 in the paper, the more stringent threshold that we chose (*E* value $1x10^{-31}$) shows how the sequences often assigned in the literature to the canonical Theta class mostly separate cleanly from those assigned in the literature to the Delta and Epsilon classes. Those Theta class proteins that are mixed in with Delta and Epsilon class proteins may thus represent sequences historically misassigned to the Theta class. Here, the inclusion of all nodes in the visualized full network for this Level 2 subgroup shows the relatively close relationship of these three classes, with Delta and Epsilon proteins being especially close. (Note: we defined the $1x10^{-31}$ threshold using Swiss-Prot annotations corresponding to canonical classes, which did not include Delta or Epsilon classes.) However, as can be seen here, this threshold results in good separation of these classes, although the annotations were extracted manually from the literature for Figure 7 after the threshold was chosen. Further analysis of other full networks of Level 2 subgroups suggests that this threshold may be generally useful for cytGST classification work (not shown).

To aid in choosing the $1x10^{-31}$ threshold, we developed a metric (shown below) to estimate the threshold at which representative network clusters correlated best with annotations. For each canonical class, we identified the single largest cluster in the network with annotated nodes in that class and marked it as the "maximum cluster for class X," with X representing the specific class being considered. This "rewarded" the level at which annotated nodes were assigned to the largest clusters and penalized (by non-counting) clusters that fragmented the classes. Then we looked to see if any of the marked clusters were marked for more than one class. If so, only the nodes for the class with the maximum number of nodes in the cluster were counted. This penalized the clustering of separate classes together. The number of counted nodes was summed for the entire network and the total count (called "sum_maxcorrbyspfam" below) was an indication of how well the clustering captured the class assignments. The procedure was iterated over a range of *E*-values by creating network clusters from $1x10^{-10}$ to $1x10^{-40}$ and calculating the metric at each *E*-value. As mentioned in the main text, we also applied the MCL method to create clusters and again calculated the metric but this time for MCL cluster assignments ("sum_maxcorrbymcl") over the same range of *E*-values to estimate how well the network clusters correlated with MCL clusters. A summary of the data is shown below (Note: NegLogE = –log(10) *E*-value):

NegLogE	sum_maxcorrbyspfam	sum_maxcorrbymcl
10	15	266
11	15	306
12	15	319
13	25	464
14	25	534
15	25	619
16	25	641
17	25	661

18	25	737	
19	25	747	
20	26	846	
21	46	1037	
22	46	1063	
23	57	1183	
24	63	1353	
25	63	1369	
26	66	1331	
27	76	1358	
28	80	1476	
29	80	1494	
30	80	1481	
31	74	1533	*
32	74	1488	
33	73	1415	
34	73	1370	
35	73	1335	
36	73	1305	
37	72	1258	
38	69	1196	
39	64	1136	
40	63	1082	

Comparison of the results showed that the threshold of $1x10^{-31}$ produced the best correlation between our network clusters and MCL assigned clusters. We preferred this cutoff to the best one for canonical classes because the canonical classes are known to be imperfect and to populate the sequence space only sparsely. This also appeared to be a good choice because it is still close to the best cutoff for the canonical classes. Finally, we visually inspected the full networks to confirm that the groupings at this threshold looked reasonable with regard to annotated classes. However, no clustering method is perfect and, as stated in the main text, these groupings are principally meant to be used as guidelines by GST experts; to explore other issues, the networks can easily be viewed by users using different thresholds.
